# Therapeutic Potency of Nanoformulations of siRNAs and shRNAs in Animal Models of Cancers

**DOI:** 10.3390/pharmaceutics10020065

**Published:** 2018-05-26

**Authors:** Md. Emranul Karim, Kyi Kyi Tha, Iekhsan Othman, Mohammad Borhan Uddin, Ezharul Hoque Chowdhury

**Affiliations:** Jeffrey Cheah School of Medicine and Health Sciences, Monash University Malaysia, Jalan Lagoon Selatan, Bandar Sunway, 47500 Petaling Jaya, Selangor, Malaysia; karim604306@gmail.com (M.E.K.); tha.kyi.kyi@monash.edu (K.K.T.); iekhsan.othman@monash.edu (I.O.); mohammad.uddin@monash.edu (M.B.U.)

**Keywords:** RNA interference, small interfering RNA (siRNA), short hairpin RNA (shRNA), viral and non-viral carriers, cancer, gene therapy

## Abstract

RNA Interference (RNAi) has brought revolutionary transformations in cancer management in the past two decades. RNAi-based therapeutics including siRNA and shRNA have immense scope to silence the expression of mutant cancer genes specifically in a therapeutic context. Although tremendous progress has been made to establish catalytic RNA as a new class of biologics for cancer management, a lot of extracellular and intracellular barriers still pose a long-lasting challenge on the way to clinical approval. A series of chemically suitable, safe and effective viral and non-viral carriers have emerged to overcome physiological barriers and ensure targeted delivery of RNAi. The newly invented carriers, delivery techniques and gene editing technology made current treatment protocols stronger to fight cancer. This review has provided a platform about the chronicle of siRNA development and challenges of RNAi therapeutics for laboratory to bedside translation focusing on recent advancement in siRNA delivery vehicles with their limitations. Furthermore, an overview of several animal model studies of siRNA- or shRNA-based cancer gene therapy over the past 15 years has been presented, highlighting the roles of genes in multiple cancers, pharmacokinetic parameters and critical evaluation. The review concludes with a future direction for the development of catalytic RNA vehicles and design strategies to make RNAi-based cancer gene therapy more promising to surmount cancer gene delivery challenges.

## 1. Introduction

**Figure 1:**
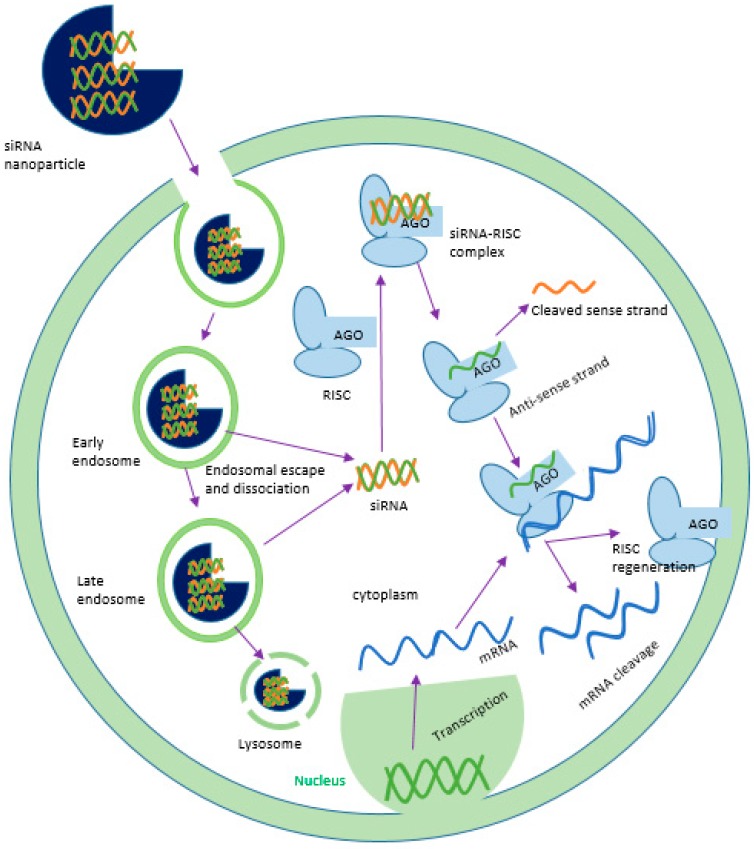
RNAi-mediated gene silencing mechanism. Cellular internalization of siRNA-nanoparticle complex via endocytosis is followed by siRNA release from both the particle and the endosome. In cytoplasm, the released siRNA is loaded into RNA-induced silencing complex (RISC), degrades the passenger strand and activates anti-sense strand. The activated anti-sense strand subsequently cleaves target mRNA. The catalytic RNAs that are unable to escape endosome are subjected to lysosomal degradation.

Gene therapy is the delivery of functional genes into the cell to modulate gene expression and the extent of their expression. Specificity and stability in biological media should be adequate to carry the corrected genetic information to their progeny for consistent effects. Since the discovery of gene sequencing technology, the journey from concept to clinical trial has faced a lot of hurdles to attain clinical reliability [1]. The era of catalytic RNA added a new dimension to the management of a broad range of critical human disorders like cancers and Alzheimer’s disease. The formal appearance of RNAi techniques based on a double stranded RNA (dsRNA) came into sight at the end of the nineteenth century to silence genes in nematodes [2,3]. The long dsRNA is able to silence gene expression in both nematodes and mammalian cells, but its non-specificity, sequence-independent pathways and activation of innate immune response make it incompatible in gene silencing technology [4,5]. At the beginning of the twentieth century, vigorous research on minimizing the shortcomings of dsRNA concluded that long dsRNAs can be processed into less than 30 base pair sequences, inducing the sequence-specific silencing of targeted genes in mammals [6]. This finding unfolded synthetic siRNA (small interfering RNA) as a new biological tool for silencing the genes in mammalian cells specifically. The smaller fragments of siRNA (21–23 nucleotides) are processed from long pieces of dsRNA by the enzyme dicer [7,8]. The siRNA can be produced synthetically and delivered into mammalian cells via multiple transfection methods. After reaching the cytoplasm of the target mammalian cell, siRNA is incorporated with RNA-induced silencing complex (RISC) [9]. Argonaute 2, a multi protein of Argonaute family is the catalytic core of RISC that unwinds the siRNA double strand and degrades the sense strand (passenger strand). The activated guide strand (anti-sense strand) containing RISC identifies and guides the sequence-specific cleavage at a position of nucleotides 10–11 of target mRNA by post-transcriptional gene silencing mechanisms, preventing translation and silencing specific gene expression [10,11] (Figure 1). The activated RISC then heads to other mRNA targets until their degradation. The degradation of activated RISC requires 3–7 days for fast growing cells and several weeks for slowly dividing cells [12,13]. On the other hand, short hairpin RNAs (shRNAs) are synthesized in the nucleus of a cell, transported into the cytoplasm and processed into siRNA for silencing target mRNA via binding with RISC. The shRNA expression vectors, therefore, need to be transported into the nucleus of a cell for transcription [14,15,16]. The precisely designed siRNA can specifically silence any gene in the body and, therefore, appears to be therapeutically more potent than conventional small molecular drugs.

**Figure 2:**
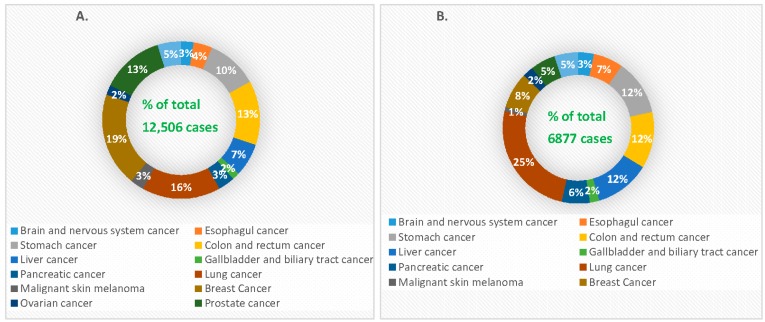
The 2015 global incidence and deaths for different type of cancers, (**A**) represents the incident rate (thousand) per 100,000 person-years; (**B**) represents the death rate (thousand) per 100,000 person-years [25].

Cancer is generally characterized by genetically abnormal signal transduction pathways that lead to heterogeneous cellular proliferation because of mutation and heritable alteration in an oncogene or tumor suppressor gene [17]. Cancer gene therapy includes gene amplification or deletion, suicide gene therapy, inactivation of oncogene by epigenetic silencing, mutation correction, chromosomal translocation, tumor suppressor activation and blocking of angiogenesis via the delivery of nucleic acids like plasmid DNA (pDNA), mRNA, short interfering or silencing RNA (siRNA), micro RNA (miRNA), short hairpin RNA (shRNA), anti-sense oligonucleotides (AONs) and DNA enzymes into the target cells [18,19,20,21]. Since the emergence of gene therapy, approximately 1300 clinical trials in different cancers have been taken place [22,23]. Cancer management still poses a major challenge with high incidence and mortality rates (Figure 2) due to the detrimental effects of conventional treatment modalities, despite the fact that the number of cancer survivors in the USA is increasing because of advancement in early cancer diagnosis and treatment [24].

Here, we review the current advancement of siRNA-based cancer therapy, emphasizing the challenges and breakthroughs of siRNA delivery especially in preclinical studies and the development of various delivery vehicles from viral vectors to inorganic nanoparticles in overcoming the biological barriers. The roles of genes in multiple cancers and the knockdown effects of those genes in tumor regression will be discussed, highlighting the pharmacokinetic parameters of the delivery vehicles in different animal models of cancers.

## 2. Challenges and Breakthrough of siRNA Delivery: From Concept to Clinical Trial

**Figure 3:**
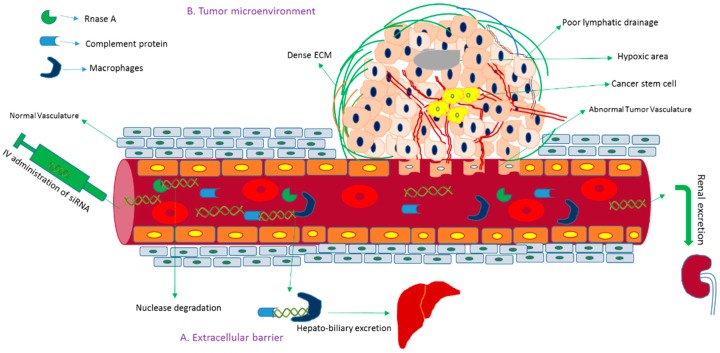
Extracellular and cellular barriers faced by unmodified siRNA to reach target site after intravenous (IV) administration. A. After IV administration, naked siRNA is subjected to extracellular barriers like nuclease degradation, macrophage-facilitated hepato-biliary excretion and rapid renal clearance. B. To enter into cancer cell, naked siRNA is to fight with cellular barriers like abnormal tumor vasculature, dense and irregular structure of extracellular matrix (ECM), high interstitial fluid pressure (IFP), hypoxia and poor lymphatic drainage, and intracellular barriers like negatively charged cell membrane and lysosomal lysis.

Gene sequencing technology has gained significant attention owing to its ability to unmask genomic data for treating disease at the genetic level. The effective and controlled intracellular delivery of siRNAs into cancer cells and cancer stem cells both in vitro and in vivo has remained a long-standing challenge, hampering siRNA-based therapeutics to move into the clinical setting. The amount of siRNA should be optimized for maximum potency. Although naked siRNA without and with chemical modification demonstrated silencing and therapeutic efficacy in the lung [26] and brain [27], respectively, following local administration, systemic administration of unmodified siRNA is subjected to nuclease degradation, phagocytic uptake, aggregation with serum protein and rapid renal clearance before reaching the cytoplasm of the target cell [28,29,30,31] (Figure 3). Once the siRNA extravasates, it faces the barriers of the dense structure of Extracellular matrix (ECM) [32], higher interstitial fluid pressure [33] and negatively charged cellular membrane. After endocytosis-facilitated entry into the cell, siRNA should escape from the endosome; otherwise, it would be subjected to lysosomal degradation [34]. Besides, naked siRNA exerts innate immune responses and off-target effects and demands repeated dosing for consistent effects [35,36,37]. To overcome these hurdles, an effective delivery vehicle is required for the delivery of siRNA to the target site by preventing the naked or modified siRNA from biological degradation and enabling it to cross the intended cellular membrane and release from the endosome [9,38,39].

**Figure 4:**
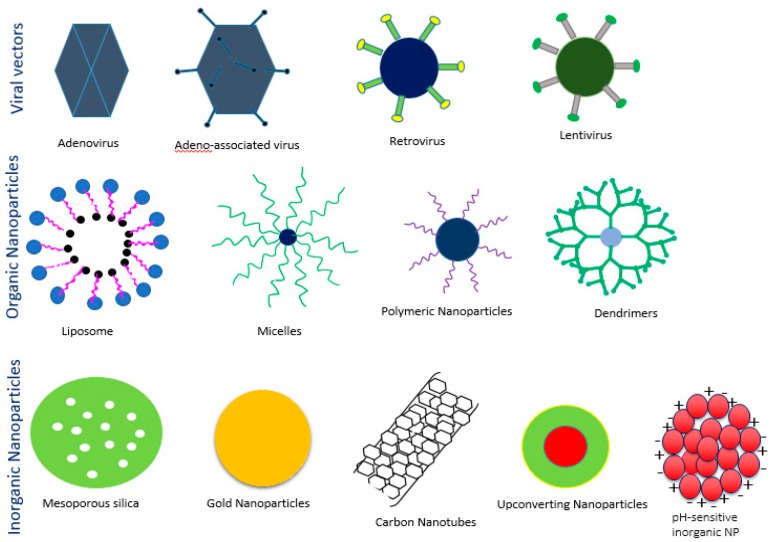
Various viral and non-viral vectors used for the delivery of genetic materials such as DNA, RNA, siRNA and shRNA.

Delivery vehicles are categorized into two main subclasses: viral vectors and non-viral vectors (Figure 4). The viral vectors like retroviruses, adeno viruses, and adeno associated virus (AAV) have been successfully used to carry genes in both preclinical and clinical trials (69% of total human trials are viral-assisted cancer gene therapy) [23]. The engineered viral vectors possess high specificity, unassisted entry to the cell membrane, lack of replication and pathogenicity and higher gene expression efficiency [40,41]. The activation of innate immune response, carcinogenicity and higher treatment cost of viral vectors limit their use in the clinic [20,40,42,43,44], thus motivating to develop non-viral vehicles for specific delivery of nucleic acids to the target cancer cells. The non-viral vectors are non- immunogenic, biologically compatible, higher specificity, higher packing capacity and less manufacturing cost in contrast to viral counterparts [23,45]. They are mainly subdivided into two categories depending on their composition: organic and inorganic carriers. The organic nanoparticles are widely used and are categorized into three classes based on their constituents, namely cationic lipids, cationic polymers and hybrids of both lipids and polymers. Several preclinical studies using lipid nanoparticles (LNPs), dendrimers, carbon nanotubes, graphene oxide and micelles, etc. as carriers have been accomplished for silencing mutant genes in multiple animal models of cancer. Among them, a number of organic nanocarriers are in clinical trials. The poor delivery efficiency, low rate of endosomal escape (1%) and retarded release of siRNA from the carriers and associated toxicity have slowed down the success rate of organic carrier-based RNAi therapy for cancers management [46,47,48]. On the other hand, inorganic nanoparticles, the newly introduced nanocarriers, have been widely explored to suppress the shortcomings of organic carriers in a gene silencing platform. Inorganic nanoparticles possess unique physicochemical properties, biocompatibility, flexibility to be functionalized with different ligands and surface-coating materials, such as Polyethylene glycol (PEG), controlled release pattern and compatibility with other existing therapeutic agents [49]. The inorganic nanoparticles that are frequently used in both in vitro and in vivo studies to repress multiple overexpressed cancer genes are gold NPs, magnetic NPs, up conversion NPs, mesoporous silica NPs, pH sensitive carbonate apatite NPs and other multifunctional NPs. Both organic and inorganic carriers have undergone several modifications like PEGylation for escaping RES (reticuloendothelial system) and renal clearance, attachment of targeting moiety like ligands and antibodies for specific delivery as part of active targeting, cell-penetrating peptides (CPP) for improving the cellular uptake and stimuli-responsive linker to improve the therapeutic index of antisense cancer gene therapy [50].

The emergence of multifunctional NPs and comprehensive investigation of RNAi against oncogenes led to potential NP-based cancer gene therapies and more than 40,000 (based on active targeting strategy) studies have been reported in the last decades [51]. However, the issues of biological stability and delivery of siRNA in the presence of multiple physiological barriers have yet to be fully resolved. Tumor microenvironment is another prominent barrier for successful intracellular delivery of RNAi therapeutics. Although the immature blood vessel with heterogeneous hyper permeability and the irregular lymphatic vasculature cause abnormal blood circulation and poor lymphatic drainage [52], respectively, resulting in enhanced permeability and retention of NPs in tumor tissues, higher interstitial pressure, synthesis of heterogeneous extracellular matrix molecules (e.g., glycoprotein) and excessive matrix metalloproteinase pose hindrance to insufficient delivery of the particles [53,54,55]. Poor cellular uptake by RNAi therapeutics is another hurdle arising as a result of repulsion between the negatively charged siRNA and the anionic cell membrane, and could be improved via chemical modification, e.g., by coating with cell-penetrating peptide [56] or stably complexing or conjugating with NPs. After entering a cell, the siRNA is usually transported from early endosome to late endosomes for degradation. To get better silencing outcome, siRNA could be released in the cytosol from endosomes at an early stage, for instance, by complexing with pH-sensitive nanoparticles (e.g., carbonate apatite). Exocytosis or cellular recycling of internalized siRNA via recycling routes e.g., non-secretory exosome might reduce the silencing efficiency of siRNAs, and could be prevented by using an exocytosis inhibitor [57,58]. Despite the successes in cell culture and preclinical studies, a small number of NPs-based siRNA formulations have successfully entered clinical trials (Table 1). Toxicity of vectors, immunogenicity and off-target effects of siRNA are the remaining hurdles in the clinic. Moreover, a difference between the physiology of humans and experimental animals, extracellular physiological barrier, technological barrier and inability to target metastases limit the success in clinical trials.

## 3. In-Vivo Delivery of siRNAs and shRNAs Directed against Different Cancer-Causing Genes in Various Cancer Models

### 3.1. Silencing of Bcl-2 Gene

**Figure 5:**
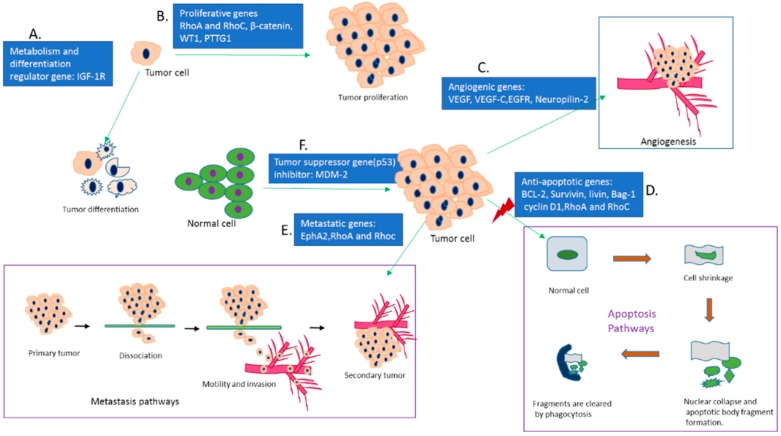
Roles of various genes in cancer development; A. Genes that regulate differentiation and metabolism of a cancer cell e.g., IGF-1R, B. Proliferative genes like RhoA and RhoC, β-catenin, WT1, PTTG1 via activating cellular process like mitosis, C. Angiogenic genes like VEGF, VEGF-C, EGFR, Neuropilin-2 that regulate tumor-induced angiogenesis, D. Anti-apoptotic genes including BCL-2, Survivin, livin, Bag-1, Cyclin D1, RhoA and RhoC which block the normal apoptosis pathways of a cell, E. Metastasis regulating genes like EphA2, RhoA and RhoC which trigger cellular motility and invasion for metastasis of tumor cell, F. Tumor suppressor gene (p53) inhibitor e.g., MDM-2, deactivates the p53 pathways.

Bcl-2 (a mitochondrial protein of 239 amino acids), is a crucial factor for promoting cell growth and survival by inhibiting apoptosis pathways of cells (Figure 5). It plays critical roles in the growth, development and metastasis of various cancers, including breast, lung, liver and gastric cancers [59,60,61,62]. Bcl-2 expression was found in 37% (10 out of 27) of biliary tract invasive cancers and the frequency of expression was observed in 90% of small cell lung cancers [63,64]. The role in cancer development and the frequency of expression make Bcl-2 as a potential target for cancer gene therapy.

The DNA vector-based Bcl-2 siRNA was synthesized under the control of H1 RNA polymerase III (pol III) promoter and mixed with liposome-protamine for evaluating anti-tumor activity of liposome-protamine-psilencer3.1 H1-Bcl-2 complexes (at a ratio of 10:3:1) in a liver cancer mouse model generated via subcutaneous injection of and H22 (liver tumor) cells into male Balb/C mice of 26 ± 2 gm. Intravenous injections of 0.4 mL of liposome-protamine-psilencer3.1 H1-Bcl-2, 0.4 mL of liposome-protamine-psilencer3.1 H1 and liposome-protamine(0.4 mL) as a control were given daily over a seven days period. There was a 66.5% decrease in tumor weight in the group of mice treated with liposome-protamine-psilencer3.1 H1-Bcl-2 in contrast to control group. Moreover, it was found that treatment with Bcl-2-silencing plasmid efficiently down regulated the expression of Bcl-2 gene in tumor cells and induced their apoptosis ratio remarkably [65]. Other researchers who used Bcl-2-targeted siRNA also observed consistent anti-cancer effects upon Bcl-2 gene silencing, indicating that the Bcl-2 antisense therapy is one of the most fruitful strategies to induce apoptosis in cancer cells [66,67].

In gallbladder carcinoma, antisense-Bcl-2-siRNA against aberrantly expressed Bcl-2 gene (positive expression rate is ~23.4–51.7%) exerted significant tumor regression effect in a mouse model study [68,69,70,71]. In an in vivo tumorigenicity study, Balb/c nude mice (4–6 weeks) of 18–22 gm were subjected to subcutaneous injection of Bcl-2-siRNA transfected human gallbladder carcinoma cells (GBC-SD) suspensions of 6 × 10^6^ cells in 0.2 mL for experimental group and GBC-SD suspensions of 6 × 10^6^ cells in 0.2 mL alone for control group into the left flank of the mice. After 39 days of treatment, it was found that the volumes of tumor for both Bcl-2-siRNA transfected group and the control group were 629 ± 78.9 mm^3^ and 1914 ± 125.0 mm^3^ respectively. The average weights of tumors were 0.77 ± 0.12 gm and 2.24 ± 0.33 gm for experimental and control groups, respectively, and the rate of tumor growth with Bcl-2-siRNA treatment was 14.99% compared to 45.58% in the control group. Thus, Bcl-2-siRNA treating mice group had three-fold reduction of tumor volume and weight in comparison to control group and significant lower tumor growth rate relative to control group [72]. For gene therapy evaluation studies, the antisense Bcl-2 sequences were mixed with recombinant plasmid vector and formed pSilencer™ EGFP sh515 (experimental group) and pSilencer™-EGFP shCon (negative control group) for comparative study. Subsequent to the establishment of a human gallbladder carcinoma model by implanting 6 × 10^6^ of GBC-SD cells (human gallbladder carcinoma cells) into the Balb/C mice of 4–6 weeks, 10 µg of pSilencer™ EGFP sh515 with 30 µL of lipofectamine-2000 liposome (Invitrogen, Carlsbad, CA, USA) (experimental group) and 10 µg of pSilencer™-EGFP shCon with 30 µL of lipofectamine-2000 liposome (Invitrogen) (negative control group) were administered into multiple sites of peritumoral tissue for five times with a two-days. After 39 days of injection, the average tumor volume for pSilencer™ EGFP sh515 containing group was approximately 700 mm^3^ (experimental group) relative to negative control group, psilencer-EGFP shcon (1400 mm^3^) and control group (1400 mm^3^), demonstrating 50% reduction of tumor volume in recombinant DNA plasmid psilencer-EGFP sh515-treated mice [72].

In addition, cationic liposome LIC-101 and B717, a sequence-specific synthetic Bcl-2-siRNA was mixed to form B717/LIC-101 for exploring tumor inhibitory effect in a prostate cancer mouse model, developed by given 5 × 10^6^ of PC-3 cells (prostate cancer cell) subcutaneously into 5 weeks old male Balb/C mice. Intravenous injections of 0.1 mg of B717/LIC-101 and 10% (*w/v*) maltose solution as a control were given for five times in a week from day 7 to day 18 after the tumor volume became palpable. The tumor volumes were measured by using Dunnett’s test at a regular time interval for 36 days of treatment. The group of mice receiving B717/LIC-101 was found to have smaller tumor volume (487 mm^3^) than the control group (1300 mm^3^), showing 63% reduction of tumor volume at the end of experiment [73].

Similarly, antitumor activity and pharmacokinetics parameter of sequence-specific Bcl-2 antisense siRNA (BO43) and liposome complex could be improved through encapsulation of PEG into the surface of liposomal–siRNA complex by enhancing blood retention time, reducing the plasma clearance by the liver and thus, increasing accumulation of siRNA in the tumor region. In a study, male Balb/c nude mice (5 weeks) were allowed to inoculate prostate tumor by injecting 3.0 × 10^6^ of PC-3 cells (human prostate cancer cells) in 100 µL of the nutrient mixture, resulting in growth of tumor size approximately 50–80 mm^3^ after 10 days of subcutaneous injection. PEGylated B043/PEG-LIC (antisense Bcl-2/PEG-LIC) was given intravenously at three different doses of 1 mg/kg, 3 mg/kg, and 10 mg/kg in two five-day consecutive courses of injections and 10% maltose solution for control groups. After 24 days of post injection, the average tumor volumes for treatments with 1 mg/kg, 3 mg/kg, and 10 mg/kg of B043/PEG-LIC were 310 mm^3^, 220 mm^3^ and 160 mm^3^, respectively, whereas for the control group it was 450 mm^3^, demonstrating the tumor inhibitory effects (65% at a dose 10 mg/kg of B043/PEG-LIC) in a dose-dependent manner. Accumulation of siRNA in S.C. tumor was also improved three-fold more than non-PEGylated liposome, resulting in superior targeted antitumor action of PEGylated siRNA-LIC complex [74].

### 3.2. Silencing of VEGF Gene

Angiogenesis is a developmental and adult physiological process of the formation of new blood vessels from preexisting micro vessels that regulate growth and metastasis of solid tumor [75,76,77,78]. Several pathological conditions like hypoxia, ischemia and inflammation result in imbalance between proangiogenic and antiangiogenic factors, leading to interactions among endothelial cells, tumor cells and a variety of growth factors. This imbalance leads to extracellular matrix remodeling, endothelial cell migration and proliferation, and capillary differentiation of tumor cells [79]. VEGF (vascular endothelial growth factor) also known as vascular permeability factor is an endothelial cell specific mitogen that plays a vital role in stimulating angiogenesis and vascular permeability through binding with VEGF tyrosine kinase receptors VEGFR1 (Flt-1), VEGFR2 (KDR, Flk-1) and VEGFR3 [80,81,82,83]. There are six subtypes of VEGF protein including VEGF-A, VEGF-B, VEGF-C, VEGF-D, VEGF-E and placental growth factor that regulate tumor-induced angiogenesis, vascular permeability and cell migration for the survival of cancer cells [84,85,86]. Over expression of VEGF and vascular density have been found to be higher in many human tumors including breast cancer and renal carcinoma, relative to normal cells [87,88,89,90,91]. Therefore, to stop metastasis of various cancer cells, VEGF gene silencing could be one of the most efficient therapeutic modalities [92,93,94].

A replication-deficient recombinant adenoviral vector-based antisense-VEGF-cDNA (Ad5CMV-αVEGF) was developed [95] and applied in an in vivo study for exploring the antitumor efficacy against a breast tumor induced by subcutaneous injection of 5 × 10^6^ of human breast cancer cells (MDA251-MB) into the mammary fat pads of athymic female nude mice (4–6 weeks of age). E_1_-deleted adenovirus type-5 without the gene (Ad5 (d1312) [96] and Ad5CMV-αVEGF were given intratumorally with 5 × 10^8^ plaque-forming units (PFU) into the mice bearing breast tumor after 4 days of inoculation. The mean tumor sizes for the Ad5CMV-αVEGF and Ad5 (d1312) were, respectively, 67.85 ± 34.65 mm^3^ and 335.23 ± 83.98 mm^3^, with 80% reduction in tumor size by the former (Ad5CMV-αVEGF) [97].

A non-viral polyplex-based delivery vehicle, composed of PEG-b-poly (_L_-lysine) di block co-polymer and PEG-grafted PEI was synthesized to deliver therapeutic genes with improved biological stability, reduced virus-associated toxicity and increased anti-tumor effects [98,99,100,101,102]. Antisense-VEGF-siRNA was encapsulated with polyplex (VEGF-siRNA-PEG) and polyethyleneimine (PEI) to form VEGF-siRNA-PEG/PEI PEC micelles to assess the tumor inhibitory effects for both intratumoral and intravenous administration in a prostate tumor model generated by subcutaneous injection of 1.5 × 10^6^ PC-3 cells (human prostate cancer cells) into the flank of the mouse. 500 pmol of VEGF-siRNA formulation at 0, 6 and 15 days was given intratumorally after tumor size grew to 50 mm^3^. Tumor regression result showed 79% reduction of tumor volume in VEGF-siRNA-PEG/PEI PEC micelles-treated mice (120 mm^3^) compared to the untreated group (590 mm^3^). For systemic delivery, 1.5 nmol of VEGF-siRNA-PEG/PEI PEC was given intravenously into the tail vein of mice at 0, 4, 10, 18 and 28 days, exerting drastic tumor inhibitory effects (86%) (tumor volume, 196 mm^3^) relative to the control group (tumor volume, 1400 mm^3^) [103].

VEGF-C, a subtype of the VEGF family is an essential lymphangiogenic factor which promotes lymphogenesis, tumorigenesis and metastasis of cancer cells [104,105,106,107]. The autocrine effect of upregulated VEGF-C initiates several intracellular signaling pathways that mediate tumor progression in various cancers like ovarian cancer, breast cancer, bladder cancer and non-small cell lung cancer (NSCLC) [105,106,107,108,109,110,111,112,113,114,115,116,117,118,119]. Hifectin was employed to deliver VEGF-C-targeted siRNA (siV2) and assess potency of the delivered siRNA against the breast tumors developed by subcutaneous administration of 5 × 10^4^ of 4T1 cells (mouse breast cancer cells) in the right-front dorsum of a Balb/C female mouse (4 weeks old) of 18–20 gm each. When the size of tumors reached at ~0.1 cm^3^, the mice were treated with siV_2_ (1 µg/gm body weight), scrambled siRNA (SCR) (1 µg/gm body weight) as a negative control and PBS (Blank control) intratumorally for every 2 days. After 3 days of post injection, the mean tumor weights for siV_2_, SCR and controls were 2.272 g, 3.242 g and 3.185 g, respectively, and the tumor volume for either control or SCR was approximately 2.5 cm^3^, whereas that for the siV_2_-treated group was 1.8 cm^3^, thus demonstrating 28% reduction of tumor volume in comparison to the control group [120].

In addition, to increase the antitumor efficacy VEGF-C-directed siRNA was mixed with lentivirus (Lv-VEGF-C-siRNA) for specific delivery to the target gene. The knockdown efficacy of virus-associated VEGF-siRNA (Lv-VEGF-C-siRNA) was assayed in a lung cancer mouse model generated via subcutaneous injection of 1 × 10^7^ A549 cells into the left flank of a Balb/c nude mouse at 6 weeks of age. Lv-VEGF-C-siRNA, Lv-siRNA negative control and PBS at a dose of 250 µL in 24 h intervals for 3 weeks into the tail vein of the mice were given after 15 days of subcutaneous injection. The average volumes of tumors for Lv-VEGF-C-siRNA-treated group and untreated group were 87.36 ± 10.93 and 241.88 ± 34.03, respectively. The weights of tumor for untreated and Lv-VEGF-C-siRNA-treated groups were 1079 ± 168.47 mg and 565.57 ± 89.33 mg, respectively, thus showing reduced tumor weight (64%) and volume (48%) in Lv-VEGF-C-siRNA-treated group relative to the untreated group. Protein and mRNA expression analysis confirmed that the silencing of VEGF-C gene expression lowered the expression of VEGFR-2, VEGFR-3, CXCR4 and CXCR7 significantly in NSCLC and suppressed the effects of AKT, ERK and p38 signaling pathways, thereby regressing the tumor growth [121]. 

Neuropilin-2 (NRP-2), a non-kinase cell surface receptor binds with VEGF and regulates vascularization and lymphogenesis of various tumors. Despite its expression in normal tissues, over expression of NPR-2 is found in colorectal cancer cells and important for the development and metastasis of colorectal cancer cells [122,123,124]. Liposomal complexes of NRP-2-targeted siRNA (NPR-2-siRNA-DOPC) could be used to slow down the tumor growth. Following administration of 1×10^6^ cells of HTC-116 cells (lenti-luc-transfected colorectal cancer cell) into the male athymic nude mice (6–8 weeks old), NPR-2-siRNA/liposomal complexes (NPR-2-siRNA-DOPC) and siRNA-DOPC (without NPR-2) as a negative control were given intraperitoneally at a dose of 5 µg of siRNA on every 5 days. The tumor volumes were measured 36 mm^3^ and 420 mm^3^ for both NPR-2-siRNA-DOPC- and control siRNA-DOPC-treated mice. Tumor growth was clearly slowed down by NPR-2-siRNA-DOPC (91.43%) compared to control siRNA-DOPC [125].

Although targeted therapeutic gene delivery has been successfully used to inhibit tumor growth by silencing specific genes, toxicity has not been diminished completely due to non-specific immune reactions and poor intracellular uptake [126,127,128,129,130]. To overcome this hurdle, self-assembled siRNA layered nanoparticles (nanoplexes) comprising PEI and PEGylated Ar3-Gly-Asp (RGD) peptide ligand were synthesized to target the tumor neovasculature expressing integrin to attenuate the VEGFR2 expression and tumor angiogenesis [131,132,133,134,135,136,137,138,139]. VEGF-R2 sequence-specific siRNA encapsulated with RGD-PEG-PEI (RPP) was subjected to an in vivo study for assessing the antitumor activity against a neuroblastoma tumor developed by administrating 1 × 10^6^ of N2A cells (mouse neuroblastoma cells) into the flank of the female nude mice (6–8 weeks old). After 7 days of inoculation, when the mice had tumor size of 20 mm^3^, the mice were treated with RPP-nanoplexes-siLacZ (nonspecific siRNA) and RPP-nanoplexes-siVEGFR-2 at a dose of 40 µg into the tail vein at every 3 days. The average tumor volume measured for VEGFR-2 sequence-specific siRNA-treated group was approximately 100 mm^3^, whereas in siLacZ-treated and control groups, the mean volumes were 1700 mm^3^ and 1200 mm^3^ respectively, demonstrating that RPP-VEGFR-2 siRNA reduced 91.67% of tumor volume in comparison to the control groups [100].

Taken together, VEGF and its subtype VEGF-C, VEGF receptors (VEGFR) and their signaling pathways NRP-2 play vital roles in developing tumor and lymphangiogenesis. The adenoviral vector-mediated siRNA reduced 80% of tumor volume in breast cancer. However, non-viral carrier, PEC loaded siRNA was shown to lower 86% of tumor volume with a minimal toxicity in prostate cancer mouse model. In case of VEGF-C, naked anti-VEGF-C-siRNA reduced only 28% of tumor volume, but when it was mixed with lentivector the inhibition rate was increased up to 48%. NRP-2 along with liposomal complex also inhibited 91.43% of tumor volume in colorectal cancer. VEGFR 2 with nanoplexes-ligand reduced 91.67% of tumor volume without any notable toxicity in neuroblastoma. The anti-tumor effects following nanoparticle-assisted delivery of tailored siRNAs directed against VEGF, NRP-2, VEGFR 2 and EGFR genes warrant further clinical investigation like toxicity to establish VEGF targeted gene therapy either alone or in combination as successful modalities for different cancer treatments. The tissue-targeted siRNA therapy allows huge improvement in silencing technology and identification of genes as the most selective targets for cancer gene therapy.

### 3.3. Silencing of EGF Receptor Genes

Epidermal growth factor (EGF) receptors, EGFR-1 and EGFR-2 are cell surface proteins that initiate growth signals in a normal cell. EGF receptors stimulate tyrosine kinase activity and activate downstream signaling pathways, playing key roles in cell division and proliferation [140]. EGFR triggers the expression of VEGF gene. Overexpression of EGFR-1 and EGFR-2 has been found in many breast cancers (30%) [141]. To improve the silencing efficacy of EGFR-1 and EGFR-2, siRNAs targeting EGFR-1 and EGFR-2 were electrostatically complexed with pH-sensitive carbonate apatite (CA) nanoparticles [142] and the anti-cancer effects of the complexes were assessed in a murine syngeneic breast cancer model. Breast tumors were inoculated with subcutaneous injection of 1 × 10^5^ 4T1 cells into the mammary pads of female Balb/c mice (15–20 g) with ages of 6–8 weeks. When the tumor volume reached 13.20 ± 2.51 mm^3^, CA-siRNA(s) against EGFR-1(50 nM) or EGFR-2 (50 nM) were given intravenously into the tail vein of mice at 3 days with a total of two injections. CA-siRNA(s) directed EGFR-1(50 nM) or EGFR-2 (50 nM) showed no robust tumor regression after 29 days of treatment. On the other hand, concurrent delivery of EGFR-1 and EGFR-2 siRNAs with carbonate apatite exerted significant tumor volume reduction (61%) in contrast to control groups. These results suggested that combined delivery of multiple siRNAs with pH-sensitive CA is a very promising strategy in treating cancer, highlighting a huge potential of pH-responsive drug delivery to the tumor microenvironment [143].

### 3.4. Silencing of Survivin Gene

Survivin is a human gene that encodes a 16.5-kD wild-type protein of 142 amino acids, which is extensively expressed at G2/M phase and declines rapidly in G1 phase of the cell cycle [144,145]. Survivin is one of the subtypes of apoptosis protein inhibitor family (IAP) including X-linked inhibitor of apoptosis (XIAP), cIAP1, cIAP2, NAIP (NLR family, apoptosis inhibitory protein), Livin, ILP2 (IAP-like protein) and BRUCE [146,147,148]. It suppresses apoptosis by blocking both intrinsic and extrinsic pathways of apoptosis [149]. Survivin can be co-immune precipitated with caspases-3, caspases-7 and caspases-9 processing and blocks apoptosis [150]. It also mediates cellular division by increasing escape from G1 checkpoint arrest and facilitates to enter into S phase subsequently and influences tumor aggressiveness in cancer patients [145,151,152,153]. Survivin helps to develop chemo-resistance against various chemotherapeutics, thereby increasing tumor recurrence rate [154,155,156,157]. Survivin expression in normal tissue is low, but an aberrant expression of Survivin is found in various types of cancers, including esophageal, lung, ovarian, central nervous system, breast, colorectal, bladder, gastric, prostate, pancreatic, laryngeal, uterine, hepatocellular, renal cancers as well as sarcoma, melanoma and hematologic malignancies [158]. This abnormal expression of Survivin makes it a potential biomolecular target for different therapeutic strategies of cancer management. Several RNAi studies targeting Survivin gene in vitro and in vivo were performed by using different carriers to deliver specifically and explore the roles of Survivin in cancer development and metastasis as well as the antitumor activity of anti-Survivin-siRNA in various cancer cell lines (Table 2).

Chitosan, a non-viral vector for gene delivery, has been widely used due to their nontoxic, non-immunogenic, biodegradable and biocompatible properties [159]. The siRNA targeted to the Survivin was electrostatically binds chitosan (CS-siRNA) for improving siRNA stabilization against nuclease-mediated degradation and avoids renal clearance [160,161]. However, a poor water-soluble CS-siRNA complex is attacked by blood proteins and attributed to protonation effects in biological media. To overcome this hurdle, the CS-siRNA complex was grafted with PEG by an ionic gelation method for increasing stability and solubility in biological media [162]. The PEG provides steric stabilization effect of CS-siRNA complex against blood proteins and cells [163,164]. Mouse breast cancer cell lines (4T1) were inoculated into female Balb/C mice at the age of 4–6 weeks, and PEG-CS-siRNA (0.3 mg siRNA/Kg), free siRNA (negative control group) and saline (blank) were administered for total five times at every 2 days after the mice had a tumor volume of 100 mm^3^. At 28 days of post-injection, the tumor volume of PEG-CS-siRNA-treated mice was found to be approximately 500 mm^3^, whereas for saline and naked siRNA treatments the volumes were approximately 1500 mm^3^ and 1100 mm^3^, respectively. PEGylated-CS-siRNA demonstrated increased biological stability and selective tumor accumulation, thereby reducing tumor growth (55%) more significantly than the non-PEGylated ones [165].

Although chitosan nanoparticles (CS-NPs) are stable in biological media, the use of CS-NPs is limited due to poor cell penetrating capability and buffering capacity. Cell penetrating peptides (CPPs), 6-poly arginine and histidine (H6R6) were combined with CS-siRNA for improvising cellular internalization and mediating early endosomal escape of antisense siRNA into the cytoplasm [166,167,168]; 6-poly arginine has strong positive charge which helps to transport of nucleic acids into tumor more easily, whereas poly-histidine increases the buffering capacity of chitosan nanoparticles and thereby destabilizes endosomal membrane in the acidic environment for rapid endosomal escape [169,170,171,172,173]. The H6R6-CS and siRNA directed to Survivin (H6R6-CS-siRNA nanoparticles) was combined by complex coacervation method [174,175] before being investigated for the activity against tumor growth and metastasis. Breast tumors were induced into 4–6-week-old Balb/C female mice via subcutaneous injection of 4T1 cells prior to the evaluation of antitumor efficacy of H6R6-CS-siRNA at a concentration of 0.3 mg/kg through injections of the formulation for five times at a two days after the tumor size became 130–140 mm^3^. Naked siRNA, as well as saline, were also given as controls. After 28 days of treatment, the tumor volume for H6R6-CS-siRNA was approximately 600 mm^3^, whereas in blank and naked siRNA-treated group the volumes were, respectively, 1600 mm^3^ and 1200 mm^3^, thus demonstrating significant antitumor efficacy for H6R6-CS-siRNA complexes (63% reduction of tumor volume) compared to the control group. The improved cellular internalization and early endosomal escape capability of H6R6-CS-siRNA complexes resulted in more transfection efficiency with higher capability of silencing Survivin gene [176].

Melanoma is one of the most common malignancies characterized by strong metastasis and chemo-resistance properties, leading to higher mortality rate relative to other cancers [177,178,179]. The combined action of anti-apoptotic factors, pro-apoptotic effectors and stringent survival signal contributes to the development of chemo-resistance. The anti-apoptotic protein Survivin has a strong correlation with melanoma metastasis and is found to be expressed at a higher level in melanoma which makes Survivin a potential therapeutic target for melanoma treatment [151,152,153]. Sticky siRNA (ssiRNA) with cationic linear polyethyleneimine (PEI) was shown to promote gene silencing by improving stability and reducing conventional toxicity [180]. Murine melanoma tumors were implanted in NMRI nude female mice (5 weeks old) by subcutaneous injection of 1 × 10^6^ B16-F10 cells into the right flank of mice. When the tumor volume grew to 50 mm^3^, the mice were treated with glucose (control), negative control ssiRNA and antisense Survivin-siRNA/PEI at a dose of 1 mg/kg. At 10 days of post injection, the tumor volumes for both ssiRNA-Survivin/PEI and control groups were approximately 1000 mm^3^ and 2000 mm^3^, respectively. The ssiRNA-Survivin/PEI reduced 50% of the tumor volume in comparison to the control group, via attenuating the Survivin gene expression [181].

Sialic acid (SA) is found to be overexpressed on cell surfaces of most cancer cells and aids in tumor metastasis by avoiding immune recognition of cancer cells [182,183,184]. Phenylboronic acid (PBA) grafted with polyethyleneimine (PBA-PEI) for targeting SA, and shielded with PEG (polyethylene glycol) to increase stability in systemic circulation, was shown to reduce off-target effects and carry anti-Survivin siRNA into targeted tumor cells. The PEG shell which could be detached from NP-siRNA complexes at extracellular pH of tumor enabled internalization of the NP-siRNA complexes into cancer cell. After internalization, the PBA-ribose ester bond was completely disrupted at the acidic pH of tumor microenvironment, releasing the siRNA into the cytoplasm. Tumor growth inhibition of the dual pH-responsive nanoparticle complex (PEG-CPB-PEI (PCPP)/siRNA) was studied in a breast tumor mouse model generated by subcutaneous injection of 1 × 10^5^ of 4T1 cells in mammary fat pad of Balb/C mice. When the mice had a tumor of 80 mm^3^, the tumor-bearing mice were treated 6 times in every 3 days with saline, naked siSUR, PEI1.8k_siSUR_, PCPP_siN.C_ (siRNA of nonsense sequences), and PCPP_siSUR_ (siRNA of anti-Survivin gene) at a dose of 1 mg/kg (siRNA). The tumor volumes of the mice treated with PCPP_siSUR_ and control groups were approximately 500 mm^3^ and 1300–1500 mm^3^, respectively, revealing notable tumor volume reduction (66%) in comparison to the control groups [185].

The rigorous study on Survivin demonstrated that Survivin is one of the key players to drive cancer formation by mediating apoptosis in various tumors. The issue of effective, non-toxic delivery and cellular internalization is a key challenge for successful siRNA delivery. To address this challenge, researchers are trying to establish a safe and secure carrier for the successful delivery of Survivin into target cancer cells. Sticky siRNA (ssiRNA) with Polyethyleneimine (PEI) reduced 50% of tumor volume in malignant melanoma. Additionally, when the siRNA was given with polyethylene grafted chitosan (PEG-CS), the tumor reduction rate increased to 55% along with reduction of side effects. Recently, to improve cellular internalization, cellular peptide proteins (CPPs) with chitosan were used to carry Survivin-siRNA which improved the antitumor efficacy (63%). Moreover, dual pH-responsive PCPP exerted significant tumor regression effects (66%) without hampering normal physiology of mice.

### 3.5. Silencing of Cyclin-B1 Gene

Cyclic-B1 is a regulatory protein, involved in mitosis. Cyclin B1, along with Ser/Thr kinase Cdc2 (cyclin-dependent kinase1, cdk1), forms the “mitosis promoting factor” and this complex triggers the cell from G2 phase to mitosis [186,187,188]. Overexpression of cyclin-B1 resulted in uncontrolled cell proliferation and hampered the stability of chromosomes [189,190,191,192,193]. Deregulated Cyclin-B1 expression has been observed in various cancers like esophageal squamous cell carcinoma, laryngeal squamous cell carcinoma, and colorectal cell carcinoma and induces resistance against radiotherapy in different tumors [194,195,196]. The silencing of the cyclin-B1 gene caused induction of growth arrest at G2 phase and stopped cancer cell division [197,198]. The inevitable role of cyclin-B1 in abnormal cellular growth makes it a striking therapeutic target for cancer management. Several studies with the siRNA targeting cyclin-B1 were accomplished in the mouse model for evaluating its role in tumorigenesis (Table 2). 

Non-covalent bonding between carrier and siRNAs is a very promising technique for complexing and delivering siRNA into tumor cells as well as embryonic stem cells [199,200,201,202,203] more efficiently. Cell-penetrating peptide, MPG-8 (Primary amphipathic peptide carrier) binds with siRNA non-covalently to form stable complexes with an siRNA which can be further functionalized for improving intracellular delivery [204,205]. The MPG-siRNA complex was tailored with cholesterol for improving stability in biological fluid and overall potency. The higher stability in biological fluid and slow release pattern of MPG-siRNA to the target tumor cells make them more advantageous over the other existing strategies. A prostate cancer mouse model was developed by injecting 10^6^ PC-3 cells into Swiss nude mice. When the mice had a tumor size of 100 mm^3^, they were treated with 0.1 mL of PBS (as a control), free cyclin-B1 siRNA (100 µg), control siRNA Cyc-B3(50 µg), cyclin-B1-siRNA (5 µg and 10 µg) complexes with MPG-8/chol-MPG-8 at a 1/20 molar ratio and cyclin-B1 siRNA with MPG-8(10 µg) in every 3 days. After 50 days of treatment, it was found that the Cyc-B1 siRNA-MPG-8/chol-MPG-8 (5 µg) reduced 60% of tumor size while for 10 µg of Cyc-B1 siRNA-MPG-8/chol-MPG-8 it was 92% in contrast to control groups. Cyclin-B1 protein expression was also reduced to 60% and 80% for both 5 µg and 10 µg of Cyc-B1 siRNA-chol-MPG-8 containing group at the end of 48 days. There was dose dependent tumor growth inhibition were seen for Cyclin-B1 siRNA treating group. The survival rate for 10 µg of Cyc-B1 siRNA-MPG-8/chol-MPG-8 was 70% at day 40 while in cholesterol free group it was only 20% [206]. On the other hand, intratumoral injection of cyclin-B1-siRNA (1 µg and 5 µg) complexes with MPG-8/chol-MPG-8 at a 1/20 molar ratio and cyclin-B1 siRNA with MPG (5 µg) caused 75% reduction of tumor volume for 1 µg and complete disappearance of tumor for 5 µg of Cyclin-B1-directed siRNA. The notable antitumor efficacy of Cyclin-B1-siRNA for both intravenous and Intratumoral administration and their prolonged survival rate confer new prospective for tumor management modality [206].

In addition, a sticky siRNA (ssiRNA) with cyclin-B1-antisense was encapsulated with cationic linear polyethylenimine (PEI) for improving target ability and biological stability. The murine melanoma tumor was inoculated into the NMR1 (5 weeks) nude mice via subcutaneous injection of 1 × 10^6^ cells of B-16-F10 into the right flank of mice. When the mice had a tumor volume of 50 mm^3^, the mice were treated with glucose (as a control), negative control ssiRNA and cyclin-B1-ssiRNA at a dose of 1 mg/kg intravenously every alternative day. After 20 days of treatment, massive enlargement of tumor volume for both control groups (1300 mm^3^ and 1100 mm^3^) were seen in contrast to cyclin-B1-ssiRNA/PEI group (700 mm^3^). The ssiRNA cyclin-B1-PEI complex delivered the cyclin-B1-ssiRNA to the targeted tumor cell and down regulated the Cyclin-B1 expression which resulted in significant inhibition of tumor growth [181].

In conclusion, Cyclin-B1 has a prominent role in tumor cell proliferation and is found to be heterogeneously expressed in multiple cancers. Sticky siRNA (ssiRNA) targeting Cyclin-B1 exerted 44% reduction in tumor volume and improved biological safety profile in malignant melanoma. However, Cyclin-B1 targeted siRNA loaded with cholesterol-layered MPG-8-CPP reduced more than 90% of the tumor volume in a dose-dependent manner. The prolonged survival rate and null immune responses rendered it a promising tool for cancer treatment in a therapeutic context for the future. 

### 3.6. Silencing of RhoA and RhoC Gene

Ras homologous A (RhoA) and Ras homologous C (RhoC) are the members of GTP/GDP-binding GTPase of the Ras superfamily [207,208]. They are low molecular weight compounds and act as molecular switches to promote cellular processes like actin and microtubule cycloskeleton organization, cell division, motility, cell adhesion, vesicular trafficking, phagocytosis, transcriptional regulation, matrix remodeling and cell mobility [209,210,211]. RhoA and RhoC are the key drivers for a series of pathologies of cancer, including cell motility, proliferation, apoptosis inhibition, cell cycle progression, invasion, metastasis and inflammation of tumor cells [212,213,214,215,216,217]. RhoA activation triggers signal transduction pathways; Rho-associated coiled coil-containing protein kinase (ROCK) activation pathway and the phosphatidylinositol-3-phospokinase/protein B (PI3-K/AKT) pathways. These activated pathways resulted cell locomotion, cell survival and expression of cell proliferative genes [218]. On the other hand, the active RhoC drives cell invasion and metastasis by increasing focal adhesion, contact information and angiogenesis [219,220]. Moreover, excessive expression of RhoA and RhoC are found to be more than 30% of all cancers which make them a vulnerable molecular target for cancer therapy.

Colorectal carcinoma is one of the fastest growing cancers all over the world [221,222,223]. There is a remarkable percentage of deaths from colorectal cancer owing to its tumor metastasis and recurrence properties [224]. Several mutant genes are responsible for proliferation, invasion and metastasis of colorectal cancer. Among them, RhoA and RhoC genes are found to be overexpressed in colorectal cancer and are considered to be potential targets for the management of colorectal cancer. Recombinant adenovirus-based shRNA-targeted RhoA and RhoC (Ad-RhoA-RhoC) were synthesized and subjected to animal studies for evaluating anti-tumor efficacy. 1.0 × 10^7^ of HCT116 (human colon carcinoma cell line) were implanted subcutaneously into the right flank of the athymic nude male Balb/C mice (15–18 g) of 4–5 weeks old. When the tumor nodules grew to 5–7 mm, the mice were assigned to treatment. Each group of mice (*n* = 7) was treated with normal saline as a control group (30 µL/mouse), Ad-Hk (negative control group) at a dose of 4 × 10^8^ pfu (30 µL/mouse) and Ad-RhoA-RhoC at a dose of 4 × 10^8^ plaque-forming unit (pfu) (30 µL/mouse) intratumorally 4 times daily at a 1-day interval of total accumulated dose of 1.6 × 10^9^ pfu (plaque-forming unit). After 17 days of treatment, the tumor volume for Ad-RhoA-RhoC was (444.38 ± 63.03) mm^3^ whereas for the control and Ad-HK groups it was (699.62 ± 190.56) mm^3^ and (678.81 ± 155.39) mm^3^, respectively. The tumor volume for control and Ad-Hk was almost five-fold higher than the starting volume. On the other hand, the Ad-RhoA-RhoC-containing group exerted a relatively slow tumor growth (2.38-fold) and reduced approximately 37% of tumor volume in comparison to the control group [225].

Aggressiveness of breast cancers is the most lethal condition that leads to death in most breast cancer patients. RhoA and RhoC are the key drivers for cancer aggressiveness e.g., increased cellular proliferation and metastasis by activating several pathways that catalyze cell survival and proliferation [207,226]. RNAi therapy targeted to RhoA and RhoC genes is supposed to be more effective to suppress tumor growth than conventional therapy. The anti-tumor effect following cytofectin-mediated delivery of anti-RhoA and anti-RhoC siRNAs was evaluated in a breast cancer mouse model, developed by employing 4 × 10^6^ of MDA-MB-231 cells (human breast carcinoma cells) into female athymic nude mice of 6 weeks of age. After 2 weeks of implantation, when the mice had a tumor volume of 20 mm^3^, the mice were treated with 100 µL of anti-RhoA (85 nM), anti-RhoC (85 nM) siRNA and cytofectin containing excipient as a control intratumarally at a 3-day interval over a period of 20 days. Mice treated with anti-RhoA siRNA (tumor volume, 200 mm^3^) and anti-RhoC siRNA (tumor volume, 600 mm^3^) showed less tumor growth than in the control group (1300 mm^3^). The angiogenesis index values of anti-RhoA and anti-RhoC siRNA were 8.75 ± 3.30 and 22.5 ± 3.32 relative to 30.5 ± 4.12 for the control group. The above findings demonstrated that anti-RhoA and anti-RhoC siRNA reduced tumor volume by 85% and 53%, respectively, in contrast to the control by down regulating *RhoA* and *RhoC* gene expression. The remarkable gene silencing effect and antitumor effects of anti-RhoA in intratumoral delivery revealed that there is a strong correlation between RhoA, and tumorigenesis. To furnish this approach for further investigation, intravenous administration of anti-RhoA siRNA is required for assessing its efficacy and safety in the systemic circulation [227].

Chitosan, a mucopolysaccharides was combined with polyisohexylcyanoacrylate (PIHCA) to deliver siRNA to the target site as well as to protect it from enzymatic degradation. The efficacy and safety profile of chitosan-polyisohexylcyanoacrylate-antiRhoA were assayed in a mouse model study. Human breast cancer (MDA-MB-231) cells (5 × 10^6^ cells in a volume of 250 µL) were injected subcutaneously into the right hind limb of the female athymic nude (nu/nu) mice aged 6 weeks to let the cells grow into a tumor of 20 mm^3^, and the mice were treated with 150 µg of siRNA/kg body weight in the low dose group and 1500 µg of siRNA/kg body weight in the high dose group, while empty chitosan nanoparticles at a 300 µg/mL were also injected as a control at every 3 days to 30 days. The anti-RhoA siRNA inhibited tumor growth in a dose-dependent manner. There was >90% decrease in mean tumor volume for 150 µg (low dose group) siRNA containing group, whereas for 1500 µg (high dose group) siRNA containing group it completely disappeared in comparison to control group [228].

Inflammatory breast cancer (IBC) is characterized by fast proliferation, metastasis, lower survival rate and local recurrences [219,229,230,231,232]. It was found that 90% of IBCs contain over-expressed RhoC GTPase gene [233] which might be involved in growth and metastasis of cancer cells. Cellular uptake and bioavailability of the anti-RhoC siRNA were increased by mixing with lipofectamine. SUM149 cells (1 × 10^7^) were injected into each female BALB/c-nu mice (47 weeks old) subcutaneously to allow them to grow into a tumor volume of 50–70 mm^3^. Intratumoral injections of saline (control), control siRNA, and anti-RhoC-siRNA (0–1 mL, 80 nM) were given at 2-day-intervals for a total of 14 days. The relative tumor volume for anti-RhoC siRNA was 3.4, whereas it was approximately 5.2 for both control and untreated siRNA, demonstrating 35% reduction of tumor volume in contrast to the control group. The survival rate after 30 days of treatment was approximately 85% for anti-RhoC siRNA-treated group, indicating that lipofectamine mediated anti RhoC siRNA had a significant effect on tumor growth reduction as well as survival rate enhancement. The down regulation of RhoC gene expression results in up-regulation of metastasis suppressor gene KAll expression as well as reducing the expression of CXCR4 and MMP cardinal regulator of breast metastasis [234].

The amazing results of pre-clinical studies (Table 2) indicate that RhoA and RhoC have different clinical roles in regulating transcriptional factors, invasion and metastasis of cancer cells in multiple cancers and are overexpressed in various solid tumors. Despite the technique’s novelty, the roles of RhoA and RhoC are being searched for successful management of cancer for further advancement in the future. Adenoviral-mediated combined delivery of RhoA and RhoC in colorectal carcinoma reduced 37% of tumor volume. RhoA-directed siRNA exerted extraordinary tumor volume inhibitory effects (85% of tumor volume) in breast cancer, whereas when it was given with chitosan the tumor volume reduction was increased to 90% (to completely abolishing) for the higher dose. On the other hand, RhoC with lipofactamine in inflammatory breast cancer reduced only 35% of tumor volume and 53% in aggressive breast cancer. Although RhoC is extensively expressed in almost all cancers, its clinical role and efficient treatment modality should be investigated for using it in the clinical setting. 

### 3.7. Silencing of β-Catenin Gene

β-Catenin is a protein that is encoded by the *CTNNB1* gene in humans and a subunit of cadherin protein, which are the key drivers for the WNT pathway [235,236]. It regulates the intracellular signal of WNT pathway and exerts a dual function, like cell adhesion and gene transcription which ultimately control the cellular proliferation [237,238,239]. β-Catenin undergoes ubiquitination and proteasome degradation without WNT pathways. However, in the presence of WNT pathways, WNT ligands prevent the β-catenin degradation by knocking down the destruction complex of β-catenin [240,241]. The activated β-catenin binds to the Tcf/Let family and initiates transcription of cyclin D1, C-myc, MMP-7, Lgr5^+^ and CD-44 in nucleus continuously without any external stimulation [242,243]. It also acts as a proto-oncogene and accumulation of β-catenin in nucleus serves as a tumor marker for diagnosis. Mutations of β-catenin have been found in a variety of solid tumors like primary hepatocellular carcinoma, colorectal cancer, breast cancer, lung cancer, cervical cancer, skin cancer, liver cancer and glioblastoma [244]. The involvement of β-catenin in the cancer development makes β-catenin as a potential molecular target for cancer gene therapy.

Overexpression of β-catenin and *APC* (adenomatous polyposis coli) genes are seen in colorectal carcinoma, mediating uncontrolled cell proliferation and development of colon cancer [245,246,247,248,249,250,251]. The elevated β-catenin levels are supposed to be lowered by introducing sequence-specific post-transcriptional silencing siRNA directed against β-catenin [252]. HCT 116 cells (colon cancer adenocarcinoma cell lines) were inoculated into female nude/nu mice (4–6 weeks old) for tumor inoculation. Oligofectamine-associated siRNA (250 pmol) targeting β-catenin and tax-siRNA (control) was given, and tumor volume and survival rates were monitored. Mice treated with antisense-β-catenin siRNA showed higher tumor inhibitory effects (three-fold smaller in tumor size) than the control group. The survival rate for the β-catenin-directed group was more significant and the expression of β-catenin was also down remarkably regulated relative to the control group [253].

The stem cells of the gastric gland (Lgr5^+^) are responsible for the development of stomach and intestines [254,255]. Mutation and dysregulation of signaling pathways of Lgr5^+^ stem cells result in intestinal cancer and gastric cancer. Cell cycle and apoptosis regulators 1 (CCAR1/CARP-1) has cell growth inhibition and apoptosis promoting effects in human breast Cancer cells [256,257]. Co-delivery of CCAR1-shRNA with β-catenin-shRNA improved the transcriptional activation of β-catenin in colon cancer cells [258]. Besides CCAR1, a coactivator of β-catenin is considered to have a vital role in the tumorigenesis and metastasis of gastric cancer. Lentivirus based Sh-CCAR1 for targeting CCAR 1 and scrambled shRNA as a negative control were prepared to explore the tumor regression capability. Male athymic nude mice of 8 weeks were taken and subjected to subcutaneous injection of 5 × 10^6^ AGS cells into the posterior leg of mice at a regular time period. After a certain time period the mice were divided into two groups (*n* = 3) and assigned to the treatment of shRNA-CCRA1 and scramble shRNA (shNuIIT) as a control. Tumor volume and width were measured found that, control group has a fast growing tumor size of 800 mm^3^ whereas CCAR1-shRNA groups has a relatively slow growing and smaller tumor size of 200 mm^3^, exerting 75% reduction of tumor size in CCAR 1-shRNA group in comparison control group [259].

In summary, the proto-oncogene β-catenin along with WNT pathway controls cellular proliferation by regulating transcriptional processes in various tumors. Overexpression of β-catenin is observed in a wide variety of cancers. β-catenin-targeted siRNA reduced 62% of tumor volume with a significant survival rate of colon cancer. Another most amazing finding was that the siRNA directed against β-catenin suppressed the β-catenin expression for a long period of time (6 h) even at a small dose, which is very promising for drug safety profile. On the other hand, for gastric cancer, shRNA directed against CCAR1 co-activator of β-catenin reduced 75% of tumor volume and lowered the growth rate. So β-catenin targeted siRNA and shRNA are very promising for further study to make it feasible to the clinical trial. 

### 3.8. Silencing of EphA2 Gene

Erythropoietin-producing hepatocellular (Eph) receptors are the tyrosine kinase receptors of the Ephrin family that play a vital role in the development of malignancy [260,261]. The Ephrin family is divided into two subclasses such as Ephrin A and Ephrin B based on their attachment to the membrane [262]. The EphA2 is located on human chromosome 1p36 [263] and contribute to develop Central nervous system (CNS) in the embryonic stage. In adult tissue, EphA2 is found to be expressed smaller amount in epithelial cells, but excessive expression almost ten-fold higher is seen in various tumors, including GBM patient tumor (61%), ovarian cancer (76%), prostate adenocarcinoma (85%), gastric cancer (77%), hepatocellular carcinoma, colorectal carcinoma and endometrial cancer [264,265,266,267,268,269,270,271,272,273]. Overexpressed EphA2 elicits oncogenic effects by enhancing cell-extracellular matrix (ECM) adhesion, anchorage-dependent growth and metastasis [274]. The expression rate, localization and significant clinical role in developing tumors make it an ideal target for cancer treatment.

The pharmacokinetic profile and specific delivery of the anti-EphA_2_ siRNA could be improved via inclusion of neutral lipid 1,2-dioleoyl-sn-glycero-3-phosphatidyl choline (DOPC) in liposome. Ovarian cancer cell lines, HeyA8 cells (2.5 × 10^5^) and SkOV3ip1 cells (1.0 × 10^6^) were given through intraperitoneal injection into female athymic nude mice (Ncr-nu). Empty DOPC liposomes, control siRNA in DOPC liposomes, EphA2-targeting siRNA in DOPC liposomes, paclitaxel (100 µg) + control EphA2 siRNA in DOPC liposomes and paclitaxel+EphA_2_ siRNA in DOPC liposomes were administered after the mice had a tumor size of to 0.5 to 1.0 cm^3^. The siRNA-liposomes at a dose of 150 µg/kg siRNA were given twice in a week and paclitaxel was given once in a week. In HeyA8 cell line, the size of the tumor for EphA_2_ targeted siRNA group was 0.98 g relative to control siRNA 1.51 g. On the other hand, in the SkOV3ip1 cell line, the tumor volume for EphA_2_-targeted siRNA and control siRNA were 0.35 gm and 0.70 gm, respectively. Liposomal anti-EphA2-siRNA reduced 35–50% of tumor size, but when it was co-delivered with paclitaxel, tumor size reduction went up to 82% relative to the control group [275].

In addition, to improve the antitumor activity of EphA2-targeted siRNA in ovarian cancer, combinatorial targeting of EphA2, Src and FAK (focal adhesion kinase) genes were made to downregulate the expression of FAK and Src (non-receptor tyrosine kinase) involved in tumor growth and aggressiveness in ovarian cancer [276,277,278,279,280,281,282]. The siRNAs-loaded DOPC liposomes were employed in ovarian cancer mouse model generated by treating female athymic nude mice with 5 × 10^5^ of HeyA8 cells and 1.0 × 10^6^ of SKOVip1 cells. The tumor-bearing mice were treated with control siRNA-DOPC, EphA2-siRNA-DOPC, EphA2+FAK-siRNA-DOPC and EphA2+Src-siRNA-DOPC intraperitoneally twice in a week at a dose of 5 µg siRNA/200 µL suspensions. EphA2-FAK-siRNA-DOPC exerted significant tumor growth reduction (90%) in comparison to EphA2-siRNA-DOPC (67%) and FAK-siRNA-DOPC (62%) in HeyA8 model. While in SkOVi31 model, there was also notable reduction of tumor growth in EphA2-FAK-siRNA-DOPC (76%)-treated group compared to the EphA2- (50%) and FAK- (61%) treated group. Combined delivery of EphA2 and FAK siRNAs decreased 67–70% of tumor weight compared to EphA2 and FAK (single) treated group. The EphA2-Src-siRNA DOPC had a less reduction rate of tumor growth than EphA2-FAK-siRNA DOPC-treated group. Besides, EphA2-FAK-siRNA-DOPC reduced 62–82% more tumor metastasis than a single group [283].

In gastric carcinoma, erythropoietin producing hepatocellular (EphA2), a receptor tyrosine kinase (RTK) is found to be over expressed in 77.3% of gastric cancer patients [260,264,266,284] and plays a vital role in metastasis via regulation of MMP-9 gene expression. MMP-9 (gelatinase-B), member of matrix metalloproteinase (MMPs) increased cancer cell metastasis by degrading denatured collagens (gelatins) and type iv collagen which are the structural components of ECM(extra cellular matrix). The MMP-9 mediated degradation of collagen is one of the most important steps in the development of cancer cell. SGC 7901 (human gastric adenocarcinoma) cells were injected into the axillary fossa region of 3–4 weeks old Balb/C mice (18–22 g), and when the tumor size grew to 52.2 ± 6.9 mm^3^, intratumoral injections of 0.9% sodium chloride as a blank control group, non-silencing siRNA with liposomes and EphA2-siRNA with liposome were given twice in a week. After 21 days of treatment it was found that EphA2-targeted siRNA inhibited the tumor growth by 43.1% compared to the negative control group. Immunocytochemistry analysis of tumor xenograft demonstrated less expression of MMP-9 in the mouse treated with EphA2-siRNA, suggesting that silencing of EphA2 gene down regulated the MMP-9 gene expression and exerted tumor inhibitory effects [285].

Briefly, EphA2 is functionally vital to tumor growth and development. It is highly expressed in a variety of cancers. In ovarian cancers, anti-EphA2-siRNA with DOPC reduced the 35–50% of tumor volume and its antitumor activity was increased when it has given with anticancer drug paclitaxel (67–82%). After couple years later, dual therapy of anti-FAK-siRNA along with anti-EphA2 in DOPC exerted significant tumor reduction (90%). The tumor metastasis rate also decreased to (62–82%) which is very promising. On the other hand, intratumoral injection of liposomal anti-EphA2 reduces 43.1% of tumor volume in gastric cancer cell which is need to be further analyzed in systemic administration to make it feasible for use in human. 

### 3.9. Silencing of MDM-2 Gene

p53, a tumor suppressor gene that is involved in cell cycle arrest, apoptosis and tumor growth inhibition. Oncoprotein mouse model minute 2 (MDM-2) inhibits the regulation of p53 tumor suppressor gene. It interacts with p53 and deactivates the p53 pathway. MDM-2 is over expressed in a various number of human tumors, including lung and prostate carcinoma [286]. Activation of p53 pathway by blocking the MDM-2 gene is considered to be an effective treatment modality of human cancers [287,288]. To attenuate the expression of MDM-2 in the tumor cell, anti-sense-MDM-2 were employed for specific tumor growth inhibition. But the delivery of siRNA in vivo is limited due to nonspecific tissue distribution, less blood circulation retention time and self-aggregation by biological salt [289]. To overcome this hurdle, pH-responsive di-block copolymer of poly (methacryloyloxy ethyl phosphorylcholine)-b-poly (diisopropanolamine ethyl methacrylate) PMPC-b-PDPA were synthesized for the delivery of siRNA for improving biological stability, tumor specific delivery and cellular uptake. The PMPC-b-PDPA/siRNA complex were prepared by precipitation method. Athymic mice (25 g) of 6–8 weeks old were taken and 5 × 10^6^ of H2009 cells (NSCLC cells) were injected subcutaneously. After ten days of injection, when the tumor size reached at 100 mm^3^, the mice were treated with; siRNA-MDM-2 loaded nanoparticles (0.32 mg/kg) and siRNA-Scr with NPS as a negative control (6.4 mg/kg) into the tail veins at 2 days for total 12 days. The volume of tumor was measured for both groups, with 67% reduction of tumor growth observed for siRNA-MDM-2-loaded nanoparticles in comparison to Scr-siRNA. The down regulation of MDM-2 might induce apoptosis by activating p53 gene, exerting a remarkable tumor inhibitory effect [290].

A mixture of siRNA(s) targeting MDM-2 gene along with other oncogenes, c-myc and VEGF that initiate and flourish the metastatic behavior of a cancer, were encapsulated with nanoparticles formulated by protamine, cationic lipid and PEG. The constructed combo siRNA-NP complex showed superior anti-tumor effects over the control. In a study, female C57B216 mice of 16–18 g (6–7 weeks old) were inoculated with 2 × 10^5^ of B16F10 cells, combo siRNA (MDM-2, c-myc and VEGF at a ratio of 1:1:1), the non-targeted NP, free siRNA and control siRNA at a dose of 0.45 mg/kg of total two consecutive doses were given. After 7 days of treatment, the mice were killed and lung of mice were collected for analysis. It was found that the control group had no notable tumor regression, whereas the combo siRNA with targeted NP exerted 20–30% reduction of tumor load. The survival rate of combo siRNA-containing group was found to be extended in comparison to control group of mice [291].

So, MDM-2 gene has a driving role in development and regulation of tumors via inhibiting expression of p53 tumor suppressor gene. MDM-2 targeted siRNA along with pH-responsive di-block copolymer of poly (methacryloyloxy ethyl phosphorylcholine)-b-poly (diisopropanolamine ethyl methacrylate) PMPC-b-PDPA reduced 67% of tumor volume in NSLSC animal model. On the other hand, combination of MDM-2, c-myc and VEGF siRNAs also reduced tumor load in lungs (20–30%) and extended survival rate were found in comparison to control group. The excellent tumor regression rate of MDM-2-siRNA and reduced lung tumor load warrant more preclinical studies (Table 2) focusing on pharmacokinetics profile and survival study to make it more reliable for clinicians. 

### 3.10. Silencing of IGF-1R Gene

IGF-1R (Type 1 insulin-like growth factor receptor) is a tyrosine kinase receptor which is the members of the insulin receptor family including IR (a homodimer), IGF-1R (homodimer), IGF-1R/R (hybrid, heterodimeric receptors) and mannose 6-phosphate receptor (IGF-2R) [292]. The insulin receptor (IR) has two subtypes IR-α and IR-β. The ligands of IGF-1R, IGF-1 and IGF-2 interact with IR receptors, resulting in receptor oligomerization, PTK activation and other pathways. The activation of these pathways mediate gene activation, DNA synthesis and cell proliferation [293,294,295]. The IGF-1R also plays a vital role in cell metabolism, differentiation, apoptosis, chemo resistance and angiogenesis as well as protecting cells from UV irradiation, cytokine and gamma radiation-induced apoptosis [296]. It is ubiquitously expressed in normal tissues for growth and multiple physiological activities, but heterogeneous expression has been found in many solid tumors like breast cancer, prostate cancer, colorectal cancer, lung cancer and hematological malignancies [297,298,299,300]. The excessive expression in tumors and their role in tumor development makes IGF-1R is an auspicious bio molecular target for cancer treatment.

Non-small cell lung cancer (NSCLC) comprises more than 80% of the total lung cancer, which is the leading cause of death all over the world with a minimal survival time [301,302,303]. Insulin like growth factor 1 receptor (IGF-1R) is expressed abnormally in lung cancer and mediates malignant transformation of lung tumor cell. Overexpressed IGF-1R gene hampered UV irradiation-, cytokine- and gamma radiation-induced cell apoptosis and play a critical role in the development of tumors [304]. Among the other treatment options, Anti-sense-siRNA targeting IGF-1R is considered due to its high specificity. Polyethyleneimine (PEI) coated anti-sense-IGF1R-siRNA were examined for assessing antitumor efficacy in human adenocarcinoma mouse model, developed by injecting 2 × 10^7^ of A549 into the male nude mice of 4–5 weeks of age. IGF-1R specific siRNA (0.125 µg/mm^3^ of tumor volume) expressing plasmids with polyethyleneimine (PEI) 3 µL/µg and negative control siRNA at a same dose were injected intratumorally 4 times every 5 days. After 40 days of treatment, the tumor volume for IGF-1R siRNA was found approximately 200 mm^3^, whereas for the control group it was approximately 490 mm^3^. The IGF-1R siRNA inhibit 60% of tumor growth in comparison to control group. The apoptotic cells were also increased remarkably in IGF-1R siRNA groups (117.6 ± 8.07) than control group (40 ± 9.11) [305].

Magnetofection is a process of delivery of nucleic acid-magnetic nanoparticles complexes to the target cells through applying magnetic field. The IGF-1R-targeted catalytic RNA was given through magnetofection for improving antitumor effects in an NSCLC animal model. Super-paramagnetic iron oxide nanoparticles (SPIONS) were complexed with cationic lipids (lipofectamine 2000) and plasmid DNA to form self-assembling magnetic lipoplexes for the targeted delivery of IGP-1R gene and evaluated the anti-cancer potential in a non-small cell lung cancer mouse model. Male Balb/CAnNcrj-nu mice of 4 weeks old were implanted with 5 × 10^6^ of A549 cells (NSCLC cells line with overexpression of IGF-1R) in 100 µL medium and allowed to grow tumor size about 400 mm^3^. The group of mice was treated with; 200 µL PBS as a control, pGFPshIGF-1R (50 µg/mouse):Lp2000 (125 µL/mouse) as a lipofection group and a pGFPshIGF-IR (50 µg/mouse) combined with MAG (50 µg/mouse):Lp2000 (125 µL/mouse) under the influence of a magnetic field (400 mT), which was holding onto the subcutaneous tumor surface for 1 min to additional 14 min following the injection. The IGF-1R-shRNA of liposomal magnetofection silenced the expression of IGF-1R 85.1 ± 3% than lipofection group 56.1 ± 6%, suggesting liposomal magnetofection improve site specificity and cellular uptake which significantly down regulated the IGF-1R gene expression [306].

Taken together, IGF-1R plays a vital role in cellular proliferation and tumor growth and aberrantly expressed in multiple cancers. The anti-sense siRNA targeting IGF-1R along with PEI reduced 60% of tumor volume and increased apoptotic cell expression more than 60% in lung adenocarcinoma. After a couple of years of this experiment, IGF-1R targeted shRNA with super-paramagnetic iron-oxide nanoparticles (SPIONs) under the influence of magnetic field silenced 86% of IGF-1R gene expression in the same lung cancer model. These exciting results provide us a novel delivery method for siRNA in different cancer cell lines.

### 3.11. Silencing of Livin Gene

Human IAP (inhibitors of apoptosis protein) are the endogenous proteins that are thwarted both extrinsic and intrinsic apoptosis pathways by interacting with specific cysteine proteases or caspases [307,308,309,310]. There are eight members of human IAP have been reported, including NAIP, c-IAP-1 (MIHB, HIAP-2), c-IAP-2 (MIHC, HIAP-1), XIAP (hILP, MIHA, ILP-1), Survivin, Apollon (Bruce), ILP-2 and Livin (ML-IAP, KIAP) [311]. Among them Livin, a 39 kDa protein is a novel family of Human IAP and it has two analogs like Livin-α (298 amino acids) and Livin-β (280 amino acids) which protect the cells of heart, placenta, lung, spleen and ovary from tumor necrosis factor (TNF) and anti-CD95-induced apoptosis [312,313]. Livin expression is limited in most of the normal tissues, but found to be overexpressed in a variety of human malignancies such as colon cancer, gastric cancer, breast carcinomas, melanomas and lung cancer [312,314,315,316]) It plays a significant role in tumor progression, chemo resistance development and anti-apoptotic activity which makes Livin a potential therapeutic target for cancer treatment. Several siRNAs targeted to Livin gene were investigated both in vitro and in vivo (Table 2) to evaluate pharmacodynamics and pharmacokinetics properties of antisense-Livin.

Transformation of non-metastatic human melanoma to metastatic melanoma and their resistance to the current chemotherapeutics account for a huge number of deaths in both male and female in U.S.A. [317,318,319,320]. The anti-Livin-siRNA was combined with single chain antibody (anti-MM scFC-tp-siRNA) for targeted delivery in order to reduce potential side effects and enhance therapeutic efficacy in vivo [321,322,323]. LiBr, malignant melanoma (MM) cells (1 × 10^7^) were given subcutaneously into right side of the flank region of a nude mouse to induce the tumor. PBS (200 µL) as a control, anti-MM scFv-tp-siRNA NC (Mock group) and anti-MM-ScFV-tp-siRNA (20 µg of siRNA) were given at 2 days through the tail vein. Anti-MM scFv-tp-Livin-siRNA(400 mm^3^) reduced 64% of the tumor volume compared to the control group (1100 mm^3^) [324].

Similarly, RNAi targeting Livin gene coated with lipofectamine were generated for improving apoptosis and chemo-sensitivity to chemotherapeutics and comparing the antitumor effects between treatment and control groups. In a human lung cancer mouse model generated by injecting 1 × 10^6^ SPCA-1 (human lung cancer cell lines), plasmid vector pS-L1, PS-NS (a non-specific sequence as a control) and pS-CMV neo plasmids (250 µg each) were injected through the intratumoral route after 7 and 14 days of post tumor implantation. The mean tumor size for control groups (Mock, pS-CMV neo or pS-NS treated) was approximately 690 mm^3^, whereas for the group treated with siRNA targeting Livin it was approximately 190 mm^3^ with 73% reduction of mean tumor size. The apoptotic fraction of siRNA-Livin group (30%) was also increased significantly compared to the control groups (5%), along with extended survival rate [325].

The above data exposed that, there was a strong correlation between Livin and tumor development. Abnormal expression of Livin is found in many solid tumors. Recently intravenous administration of antibody conjugated siRNA in Livin reduces 64% of tumor volume in malignant melanoma. But in lung cancer cells, Intratumoral administration of Livin-siRNA along with lipofectamine reduces 73% of tumor volume and increased 30% of apoptotic fraction. It should be extended to systemic administration for testing pharmacokinetics parameter for further advancement.

### 3.12. Silencing of WT1 Gene

Wilms’ tumor gene 1 (WT1) is a 52–54 kDa protein, encodes four zinc finger transcriptional factors located at chromosome 11p13q and arises due to inactivation of the WT1 alleles gene [326,327]. It is the key drivers that control cell proliferation and apoptosis via attenuation of the expression of proliferative gene [328]. WT1 was defined as a tumor suppressor gene but recent evidence claimed that, it acts as an oncogene in leukemogenesis and tumorigenesis [329,330,331,332,333,334]. It is highly expressed in most of the acute myeloid leukemia (AML) and acute lymphoid leukemia (ALL) [335,336,337], lung, breast, thyroid and melanomas [338,339,340,341]. High rate of expression of WT1 and their significant role as a prognostic factor makes WT1 mRNA an ideal tumor marker for leukemic blast cells as well as a potential target for RNAi mediated gene therapy for various cancer treatments (Table 2).

Signal transducers and activators of transcription 3 (STAT-3) enhances the expression of anti-apoptotic gene MCI-1 and BCL-XL as well as other gene like cyclin D1/D2 and c-Myc which ultimately mediates cancer development and progression [342,343,344,345,346]. Over expression of WT1 increased transcriptional activity of phosphorylated STAT 3 (P-STAT 3) that upregulated anti-apoptotic gene and regulate the growth and differentiation of lung tumor cells [338,347]. To identify the relationship between over expressed WT1 and NSCLC (non-small cell lung cancer) development as well as to reveal the antitumor activity of WT1-targeted shRNA in vivo, NSCLC model was generated by subcutaneously injecting 5 × 10^6^ cells into Balb/C ^nu/nu^ mice. When the tumor size reached at palpable size, the three groups of mice were subcutaneously treated with pLV-GEP-WT1, pLL3.7-WT1-shRNA (plasmid expressing WT1-shRNA) with wild type WT1 and empty plasmid (as a control). After 31 days of treatment, remarkable reduction of tumor volume was found for pLL3.7-WT1-shRNA (76%, 74% and 69%), whereas tumor growth curves for pLV-GEP-WT1 and the control group were found to be increased [348].

Transferrin (Tf), a glycoproteins over expressed on the surface of the cancer cells was used as a ligand to carry WT1-shRNA to tumor [349,350,351,352]. The WT1 shRNA-Tf was complexed with liposomes and polyethylene glycol (PEG) for improving biological stability of the shRNA in the systemic circulation. To explore the antitumor activity, B16F10 cell (5 × 105 cells) were subcutaneously injected into the female C57BL/6 mice (20–25 g) of 7–8 weeks aged, prior to intravenous administration of 50 µL of Lip-RNAi-Tf, lip-RNAi against WT1 (50 µL), 50 µL of lip-GFP-Tf (PEGFP-N3 vector, liposome and Tf complex) and 50 µL of saline solution at a 5 days until 29 days. The mean tumor weight for Lip-RNAi-Tf was 5.5 g whereas it was 8.8 g for untreated group. The mice of the other two groups were not counted because of all of the mice were dead after day 30. The liposome-WT1-shRNA-Tf reduced 34% of tumor weight in comparison to control group. Survival rate was also prolonged in liposome-WT1 shRNA-Tf containing group (62.5%) than control group (22.2%) [353].

In summary, WT1 plays key role in leukemogenesis and tumorigenesis as an oncogene but it was originally known as a tumor suppressor gene. Overexpression of WT1 has been detected in many solid tumors. In different lung cancer cell lines (A549, H1299 and H1650), anti-WT1 shRNA exerted notable tumor reduction 76%, 74% and 69% for lung cancer cell lines. On the other hand, ligand conjugated WT1-shRNA reduces 34% of tumor weight with prolonged survival rate. So this variation arises more questions about the role of WT1 and their targeting efficacy for the management of different tumors. To go for clinical setting more investigation is required to establish WT1 as a potential target for cancer management. Although clinical role of WT1 is obscured, liposome and ligand mediated delivery reduced the secondary effects and increased site specific delivery, which is very promising for the delivery of catalytic RNA.

### 3.13. Miscellaneous

The human Bcl-2-associated athanogene-1 (Bag-1) encodes the three major isoforms, including Bag-1S (p36), Bag-1M (p46) and Bag-1L (p50) that are located on chromosome 9 and involved in differentiation, cell cycle and apoptosis. It regulates Bcl-2 gene expression and mimics the anti-apoptotic activities via bridging between the growth factor and anti-apoptotic mechanism. The aberrant expression of Bag-1 has been found in breast, lung, cervix, esophagus and colorectal cancers [354,355,356]. Colorectal cancer has the second largest morbidity and mortality rate all over the world [357,358] and human Bcl-2 associated athanogene-1(bag-1) anti-apoptotic gene is found to be involved in the tumor genesis of colorectal cancer (CRC) by mediating progression and metastasis and acts as a positive regulator of Bcl-2 gene in CRC [359]. The anti-Bag-1-siRNA plasmid was combined with magnetic gold nanoparticles and evaluated its tumor inhibitory effects in colon cancer mouse model, developed by giving 1 × 10^6^ of LoVo cell (human colon cancer cell lines) into the right flank of the Balb/C nude mice (14–16 g) of 4–5 weeks old. The tumor-bearing mice were divided into five groups and treated with; normal saline, nanoparticles (25 µg), plasmid (5 µg), nano-plasmid complexes (nano-plasmid-1; 5 µg plasmid/25 µg nanoparticles) and nano-plasmid complexes (nano-plasmid 2; 5 µg plasmid/25 µg nanoparticles) intratumorally. Nano-plasmid 2 was given to mice under the influence of about 5000 gauges magnetic fields. The 5 doses were given at a 3 day intervals. The tumor volume was measured after every 3 days, 28 days later of treatment, the tumor volume for nano-plasmid complex-1 was found to be approximately 200 mm^3^, whereas it was 700 mm^3^ and 650 mm^3^ for nanoparticle alone and control group and it was 69% reduction of tumor volume. The nano-plasmid-2 under the influence of magnetic field reduced the tumor volume at a same rate of nano-plasmid-1 without any significant reduction. Silencing of Bag-1 gene was supposed to down-regulate the expression of C-MYC protein that is clinically important for colorectal cancer. The potent anti-tumor effect makes anti-Bag-1-siRNA as a potential therapeutic for colorectal cancer management [360].

The pituitary tumor transforming gene 1 (PTTG1) is the member of PTTG family, including PTTG2 and PTTG3, that plays a vital role in several cellular processes like mitosis, DNA repair, apoptosis and gene regulation. Abnormal expression of PTTG-1 interferes in cellular processes and causes aneuploidy which is the necessary events in the tumorigenesis [361,362,363,364,365]. PTTG1 is over-expressed in various cancers and promotes tumor development and angiogenesis via triggering the expression of the fibroblast growth factor 2 and VEGF [366,367,368,369,370,371]. Tuning of PTTG1 gene expression by using anti-sense siRNA is considered to be an effective modality to reduce cancer aggressiveness. Hepatocellular carcinoma (HCC) is the most prominent tumor in humans all over the world having a higher alarming rate of incidence rate [372,373]. Over expression of PTTG-1 hampered the expression and function of intact p53, which ultimately promotes hepatocellular carcinogenesis [374].

Adenovirus vector assisted delivery of siRNA targeting PTTG-1 would be more efficient in down-regulation of PTTG-1 expression for hepatocellular carcinoma management. Five weeks old Balb/C nude mice were given with SH-J1 cells (10^7^) in 100 µL of PBS subcutaneously into the right flank of the mice and allowed to grow to a tumor volume of 3–5 mm in diameter. Following intratumoral administration of 1 × 10^9^ plaque-forming units (pfu) of Ad-PTTG-1-siRNA into the mice, tumor regression study demonstrated that Ad-PTTG-1-siRNA-treated groups regressed 84% of tumor volume in comparison to the control groups, suggesting that Ad-PTTG1-siRNA may serve a new paradigm for treating human cancers [375].

CD-47 is a membrane receptor of the immunoglobulin (Ig) superfamily that induces the phagocytic process of macrophages via binding with signal regulatory proteins α (SiRP α). Overexpression of CD-47 causes the tumor cells escape from immunosurveillance and results in tumor progression [376,377]. The expression level of CD-47 was reported to be higher in leukemic cell lines, bladder-tumor-initiating cells and lymphomas [378,379,380,381]. The LPH-NPs (liposome-protamine-hyaluronic acid) were constructed to carry CD-47-targeted siRNA for evaluating pharmacodynamic properties against melanoma tumors. C57B2/6 mice of 6–8 weeks were harvested with subcutaneous injection of B16F10 cells at a concentration of 2 × 10^5^ cells/50 µL into the hind legs of mice. The mice were treated with 12 µg of CD-47 or control siRNA (0.6 mg/kg) along with LPH-NPs intravenously after the mice had a tumor volume of 50 mm^3^. The doses were given at a one-day interval to a total of six injections from the 8th day of tumor implantation. The tumor volume for CD-47-siRNA-LPH-NPs was found to be smaller than the control group, with approximately 93% reduction of tumor volume in comparison to the untreated group [382].

## 4. Future Directions

In preclinical studies, catalytic RNAs are mixed with several viral and non-viral vectors for safe, effective and targeted delivery to the cytoplasm of cancer cells. As exemplified in this review through various in vivo studies, siRNAs or shRNAs targeted to oncogenes, tumor suppressor genes and anti-angiogenic genes have been used with the help of various carriers over the past two decades for improving anticancer efficacy (Table 2). Among them, only few are translated into human trials and the others are in the pipe line. Although tremendous progress has been made, limitations still remain in the systemic applications of RNAi-based cancer nanotherapeutics. These hurdles should be addressed and resolved for the rational design of delivery vehicles for the targeted drug delivery to cancer cells in the future. Firstly, the issues of manufacturing hurdles like small size distribution, homogeneity, uniform functionalization, reproducibility at a large scale and manufacturing cost. Secondly, the poor understanding of mechanistic behavior of RNAi therapy with a view to their penetration and deposition in tumor tissue are not still clear. Thirdly, the poor cellular uptake and lower silencing effects which are the key parameters of therapeutic index along with less tumor accumulation (0.7%). These obstacles have hampered the progress of RNAi-based cancer therapy to get clinical approval.

**Figure 6:**
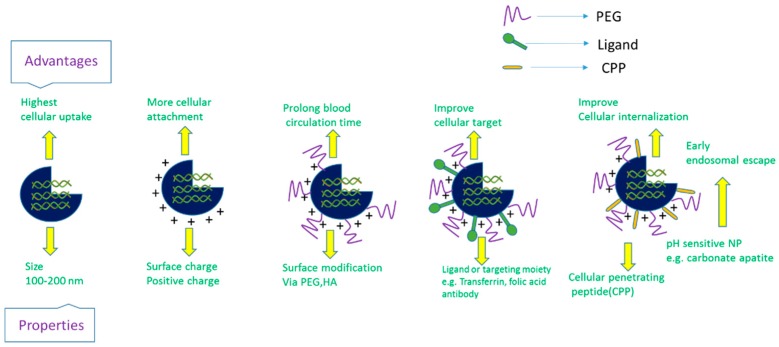
A proposal of a tailored made SMART nanocarrier (S = specific size; M = modified surface chemistry; A = accelerated cellular internalization; R = rapid endosomal escape T = targeted tumor cells).

To minimize the hurdles mentioned above, extensive research on molecular events of cancer pathogenesis and mechanisms of siRNA delivery including cellular attachment, target binding, biological interactions, intracellular trafficking and nuclease attack should be further studied to understand absolute biological phenomena. This new RNAi-based therapeutic entity should overcome the physiological barriers and differentiate between cancer and normal cells for a broader therapeutic index. From a clinical standpoint, biological readouts, bio distribution and kinetics should be analyzed to ensure safety and efficacious therapies. Future studies should, therefore, be emphasized for development of SMART nano carrier (Figure 6) and computer assisted drug delivery for evaluating pharmacokinetics and pharmacodynamics profiles before heading to clinical trials.

## 5. Conclusions

Although RNAi technology has advanced rapidly and become a clinical reality as this technology has the capability to reach and treat cancer at the molecular level, cancer target discovery and validation, gene editing system and cancer stem cell targeting demand further research for improved RNAi-based cancer therapy. The RNAi-based cancer therapy would, therefore, lead the next wave of cancer nanomedicine.

## Figures and Tables

**Table 1:** siRNAs-based clinical trials for cancer therapy.

Drug Formulation	Target Gene	NPs	Treatment	Diseases	Phase	Status	Identifier Trial Number (https://clinicaltrails.gov)
DCR-MYC	MYC	Lipid	siRNAs	Hepatocellular carcinoma	1/2	Ongoing, not recruiting 2014-present	NCT02314052
DCR-MYC	MYC	Lipid	siRNAs	Solid tumors, multiple myeloma, non-Hodgkin lymphoma, or pancreatic neuroendocrine tumors	1	Ongoing, not recruiting 2014-present	NCT02110563
ALN-VSP02	KSP and VEGF	Lipid	siRNAs	Solid tumors	1	Completed	NCT00882180
Atu 027	PKN3	Lipid Nanoparticles	siRNAs	Advanced cancers	1	Completed	NCT00938574
TKM-080301	PLK1	Lipid	siRNAs	Primary and secondary liver cancer	1	completed	NCT01437007
	PLK1	Lipid	siRNAs	Neuroendocrine tumors	1/2	completed	NCT01262235
	PLK1	Lipid	siRNAs	Advanced hepatocellular carcinoma	1/2	completed	NCT02191878
siRNA-EphA2-DOPC	EphA2	Lipid	siRNAs	Advanced solid tumors	1	Recruiting	NCT01591356
siG12D-LODER	KRAS	LODER polymer	siRNAs	Ductal adenocarcinoma or pancreatic cancer	1	completed	NCT01188785
siG12D-LODER	KRAS	LODER polymer	siRNAs	pancreatic cancer	2	Not yet recruiting	NCT01676259
SNS01-T	eIF5A	polyethyleneimine	siRNAs plasmids	Multiple myeloma	1/2	unknown	NCT01435720

**Table 2:** Summary of animal studies of siRNA- and shRNA-based cancer gene therapy.

Target Genes	Role of Genes	Delivery Vehicle	Treatment	Preclinical Studies Application	Preclinical Studies Outcome	Refs.
Bcl-2	Inhibits apoptosis pathways and promote cellular growth and survival in breast, lung, liver and gastric cancer	Liposome-protamine	siRNA	Balb/c mouse model inoculated with H22 liver tumor cells	66.5% reduction of tumor growth by suppressing Bcl-2 gene expression	65
Bcl-2		pSilencer^TM^-EGFP sh515	shRNA	Balb/C mouse model inoculated with GBC-SD, gallbladder carcinoma cells	50% reduction of tumor volume and decreased tumor growth rate	72
Bcl-2		Cationic liposome, LIC-101	siRNA	Balb/C nu^++^ mouse model inoculated with PC-3 prostate cancer cells	63% reduction of tumor volume	73
Bcl-2		PEG-LIC complex	siRNA	Balb/C mouse model inoculated with PC-3 human prostate cancer cells	Increased siRNA uptake, 65% tumor reduction without any systemic toxicity	74
VEGF	Stimulates angiogenesis and vascular permeability	Adenoviral vector (Ad5CMV)	siRNA	Athymic female mouse model inoculated with MDA251-MB, human breast cancer cells	Reduced 80% of tumor through anti-angiogenesis mechanism	97
VEGF		Polyelectrolyte complex (PEG/PEI PEC micelles)	siRNA	Female nude mice (nu/nu) model inoculated with PC-3 human prostate cancer cells	Intratumoral injection caused 79% tumor inhibition; Intravenous administration reduced 86% of tumor volume	103
VEGF-C	Promotes lymphogenesis, tumorigenesis and initiates metastasis	Hifectin-mediated transfection	siRNA	Balb/C mouse model inoculated with 4T1 cells, mouse breast cancer cells	Reduced 28% of tumor volume.	120
VEGF-C		Lentivirus vector (Lv)	siRNA	Balb/C mouse model inoculated with A549, human NSCLC cells	64% tumor inhibition and 48% reduction of tumor volume by decreasing VEGF-C expression	121
NRP-2	Binds with VEGF and regulates vascularization and lymphogenesis of various tumors	DOPC (neutral lipid 1,2-dioleoyl-sn-glycero-3-phosphatidyl choline)	siRNA	Male athymic nude mouse model inoculated with HTC-116, human colorectal carcinoma cell lines	Reduced 91.3% of tumor volume via increasing anti-angiogenic mechanism	125
VEGF R2	Regulates angiogenesis and tumor growth	RGD (Ar3-Gly-Asp peptides)-PEG-PEI nanoplexes	siRNA	Female nude mouse model inoculated with N2A, mouse neuroblastoma cells	Enabled tissue-specific delivery and inhibited more than 90% of tumor volume	100
EGFR 1 & ERBB2	Activate downstream signaling pathways and play key role in cell division and proliferation	Carbonate apatite Nano-particle	siRNA	Female Balb/C mouse model inoculated with 4T1 cells, mouse breast cancer cells	61% reduction of tumor volume without any toxicity	143
Survivin	Suppresses apoptosis by inhibiting both intrinsic and extrinsic pathways of apoptosis, as well as improves chemo-resistance to various chemotherapeutics and increases tumor recurrence rate	PEGylated chitosan (PEG-CS)	siRNA	Female Balb/C mouse model inoculated with 4T1 cells, mouse breast cancer cells	Increased biological stability and targeted gene delivery, reduced 55% of tumor volume	165
Survivin		Chiosan-6-poly arginine and histidine (H_6_R_6_-CS)	siRNA	Female Balb/C mouse model inoculated with 4T1 cells, mouse breast cancer cells	Improved cellular uptake and endosomal escape with 63% tumor inhibition	176
Survivin		Cationic linear polyethyleneimine (PEI)	Sticky siRNA (ssiRNA)	NMRI nude female mouse model inoculated with B16-F10 cells, murine melanoma cell lines	Reduced 50% of tumor volume through silencing of Survivin gene	181
Survivin		PCPP (PEG-CPB-PEI) nano-particle	siRNA	Balb/C mouse model inoculated with 4T1 cells, mouse breast cancer cells	Increased tumor accumulation and improved cellular uptake with 66% reduction of tumor volume	185
Cyclin-B1	As a mitosis promoting factor triggers uncontrolled cell proliferation and hampers the stability of chromosomes	MPG-8 (Primary amphipathic peptide carrier)-cholesterol (MPG-8/chol)	siRNA	Swiss nude mouse model inoculated with PC-3 cells, human prostate cancer cells	90% tumor size inhibition for maximum dose, 60–80% reduction of Cyclin B1 expression and extended survival rate	206
Cyclin-B1		Cationic linear polyethyleneimine (PEI)	Sticky siRNA (ssiRNA)	NMRI nude female mouse model inoculated with B16-F10 cells, Murine melanoma cell lines	Reduced 44% of tumor volume via down regulating Cyclin B1 expression	181
RhoA & RhoC	Triggers signal transduction and drives a series of pathologies of cancer including cell motility, proliferation, apoptosis inhibition, cell cycle progression, invasion, metastasis and inflammation	Adenoviral vector	shRNA	Male Balb/C mouse model inoculated with HTC-116, human colorectal carcinoma cell lines	Slowed tumor growth (2.38 fold) and reduced 37% of tumor volume	225
RhoA & RhoC		Cytofectin-mediated transfection	siRNA	Athymic female mouse model inoculated with MDA251-MB, human breast cancer cells	Reduced tumor volume 85% (anti-RhoA) and 53% (anti-RhoC), lowered angiogenesis index	227
RhoA		Chitosan-PIHCA (polyisohexylcyanoacrylate)	siRNA	Athymic female mouse model inoculated with MDA251-MB, human breast cancer cells	At higher dose the tumor were completely removed	228
RhoC		Lipofectamine-mediated transfection	siRNA	Balb/C-nu mouse model inoculated with SUM149, human IBC cells	Reduced tumor volume by 35%, increased survival rate to 85%, up-regulated metastasis suppressor gene KAll	234
β-Catenin	Regulates cell-cell adhesion and gene transcription, ultimately controlling cellular proliferation	Oligofectamine-mediated transfection	siRNA	Female nude/nu mouse model inoculated with HTC-116, human colorectal carcinoma cell lines	Three-fold smaller in size of tumor in comparison to control with extended survival rate	253
β-Catenin		Lentivirus vector	shRNA	Male athymic nude mouse model inoculated with AGS cells, human gastric cancer cells	75% reduction of tumor volume by inhibiting CCAR1 gene expression	259
EphA2	Enhances cell-extracellular matrix (ECM) adhesion, anchorage-dependent growth and metastasis	DOPC (neutral lipid 1,2-dioleoyl-sn-glycero-3-phosphatidyl choline)	siRNA	Female athymic nude (Ncr-nu) mouse model inoculated with SkOV3ip1 cells, ovarian cancer cell lines	Reduced 35–50% of tumor size	275
EphA2 and FAK		DOPC (neutral lipid 1,2-dioleoyl-sn-glycero-3-phosphatidyl choline)	siRNA	Female athymic nude (Ncr-nu) mouse model inoculated with SkOV3ip1 cells, ovarian cancer cell lines	Reduced 62–82% of tumor metastasis and slowed down tumor growth rate	283
EphA2		Liposome	siRNA	Balb/C mouse model inoculated with SGC 7901, human gastric adenocarcinoma cells	43.1% inhibition of tumor growth, with reduction in expression of metastatic gene MMP-9	285
MDM-2	Inhibits the regulation of p53 tumor suppressor gene	PMPC-b-PDPA (di-block copolymer of poly (methacryloyloxy ethyl phosphorylcholine)-b-poly (diisopropanolamine ethyl methacrylate)	siRNA	Athymic mouse model inoculated with H2009 cells, NSCLC cells	67% reduction of tumor growth via down regulation of MDM-2 gene expression without any systemic toxicity	290
MDM-2, c-myc and VEGF		cationic lipid-PEG	siRNA	Female C57B216 mouse model inoculated with B16-F10 cells, murine melanoma cell lines	20–30% reduction of tumor load with extended survival rate	291
IGF-1R	Promotes cellular metabolism, differentiation, apoptosis, chemo resistance and angiogenesis as well as protecting cells from UV irradiation, cytokine and gamma radiation-induced apoptosis	Plasmids-PEI	siRNA	Male nude mouse model inoculated with A549 cells, human lung adenocarcinoma cell lines	60% of reduction of tumor volume, Increasing apoptotic cells	305
IGF-1R		Magnetic lipoplexes	shRNA	Male Balb/C AnNcrj-nu mouse model inoculated with A549 cells (NSCLC cells line with overexpression of IGF-1R)	Improved site specificity and cellular uptake, and reduced 85.1 ± 3% of IGF-1R gene expression	306
Livin	Thwarts both extrinsic and intrinsic apoptosis pathways by interacting with specific cysteine proteases or caspases, while playing a significant role in tumor progression and chemo resistance development	Single chain antibody	siRNA	Nude mouse model inoculated with LiBr cells, malignant melanoma cell lines.	Reduces approximately 64% of tumor size.	324
Livin		Plasmid vector	siRNA	Balb/C nu/nu mouse model inoculated with SPCA-1 cells, human lung cancer cell lines.	73% reduction of mean tumor size, with increased apoptotic fraction and improved survival rate	325
WT1	The key drivers that control cell proliferation and apoptosis via regulation of the expression of proliferative genes	Plasmid vector	shRNA	Balb/C ^nu/nu^ mouse model inoculated with A549, H1299 and H1650 cells	69–76% reduction of tumor volume without any systemic toxicity	348
WT1		Liposome-PEG	shRNA	Female C57BL/6 mouse model inoculated with B16F10 cell, murine melanoma cell lines	Reduced 34% of tumor weight and extended survival rate (62.5%)	353
Bag-1	Regulates Bcl-2 gene expression and mimics the anti-apoptotic activities via bridging between the growth factor and anti-apoptotic mechanisms	Magnetic gold nanoparticles	siRNA	Balb/C nude mouse model inoculated with LoVo cell, human colon cancer cell lines	69% of tumor inhibition without toxicity	360
PTTG1	Plays a vital role in several cellular processes like mitosis, DNA repair, apoptosis and gene regulation and causes aneuploidy.	Adenoviral vector	siRNA	Balb/C nude mouse model inoculated with SH-J1 cells, hepatoma cell lines	Significant tumor inhibition efficacy (84%)	375
CD-47	Causes the tumor cells escape from immunosurveillance with the result of tumor progression	Liposome-protamine-hyaluronic acid	siRNA	C57B2/6 mouse model inoculated with B16F10 cell, murine melanoma cell lines	Above 90% tumor inhibition without toxicity	382
